# A Review of Polymer-Based Materials for Fused Filament Fabrication (FFF): Focus on Sustainability and Recycled Materials

**DOI:** 10.3390/polym14030465

**Published:** 2022-01-24

**Authors:** Daniela Fico, Daniela Rizzo, Raffaele Casciaro, Carola Esposito Corcione

**Affiliations:** 1 Dipartimento di Ingegneria dell’Innovazione, Università del Salento, Edificio P, Campus Ecotekne, S.P. 6 Lecce-Monteroni, 73100 Lecce, Italy; daniela.fico@unisalento.it; 2 Dipartimento di Beni Culturali, Università del Salento, Via D. Birago 64, 73100 Lecce, Italy; daniela.rizzo@unisalento.it (D.R.); raffaele.casciaro@unisalento.it (R.C.)

**Keywords:** additive manufacturing, polymers, sustainability, fused filament fabrication, cultural heritage

## Abstract

Recently, Fused Filament Fabrication (FFF), one of the most encouraging additive manufacturing (AM) techniques, has fascinated great attention. Although FFF is growing into a manufacturing device with considerable technological and material innovations, there still is a challenge to convert FFF-printed prototypes into functional objects for industrial applications. Polymer components manufactured by FFF process possess, in fact, low and anisotropic mechanical properties, compared to the same parts, obtained by using traditional building methods. The poor mechanical properties of the FFF-printed objects could be attributed to the weak interlayer bond interface that develops during the layer deposition process and to the commercial thermoplastic materials used. In order to increase the final properties of the 3D printed models, several polymer-based composites and nanocomposites have been proposed for FFF process. However, even if the mechanical properties greatly increase, these materials are not all biodegradable. Consequently, their waste disposal represents an important issue that needs an urgent solution. Several scientific researchers have therefore moved towards the development of natural or recyclable materials for FFF techniques. This review details current progress on innovative green materials for FFF, referring to all kinds of possible industrial applications, and in particular to the field of Cultural Heritage.

## 1. Introduction

Additive manufacturing (AM), also known as three-dimensional (3D) printing, is a class of promising machineries that produce objects starting from computer-aided design (CAD) models, by adding materials in a layer-by-layer style [1,2,3].

The layer-by-layer method allows the creation of complex geometries, such as topologically optimized, integrated and functional parts with minimum material wastage and reasonable speed [4,5]. According to the ISO/ASTM 52900:2015 standard, there are seven types of AM systems, including material extrusion (ME), material jetting (MJ), binder jetting (BJ), sheet lamination (SL), vat photo polymerization (VP), powder bed fusion (PBF) and directed energy deposition (DED) [3]. Each AM method has its own characteristics in speed, resolution and costs, thus presenting different options for customers [6]. ME, also known as fused filament fabrication (FFF) is the most commonly-used AM technique which includes selective deposition of thermoplastic polymer through a heated nozzle. The melted polymer is extruded onto a build stage to form predetermined thin layer and further solidifies and bonds together with neighbor layers to produce a part with dimensional accuracy on the order of 100 μm [3,7]. At the moment, polymer components manufactured by FFF method can achieve the requirements of many applications, such as toys, textiles, daily life [8], flexible microfluidic and strain sensors in electronic area [9,10,11], further to customized implants and scaffolds in biomedical area [12,13,14,15,16,17,18,19,20], automotive and aerospace [21,22], prototypes for functional testing, lightweight component [23,24] and cultural heritage restoration. Nevertheless, the growth of FFF from a prototyping technique into a manufacture apparatus is delayed by numerous questions, such as poor surface quality determined by nozzle dimensions and polymer viscoelasticity [25,26], low build speed [2,27] and limited material selection relative to those for conventional manufacturing procedures [28]. In order to enhance the mechanical characteristics of the 3D printed objects, several polymer-based composites and nanocomposites filaments for FFF have been developed and experimentally characterized. Nevertheless, some of the proposed innovative materials with enhanced mechanical properties are not biodegradables. Consequently, their waste disposal represents a crucial matter, that requires an immediate answer. The most promising way to solve this environmental problem is to explore the possibility to use sustainable materials, according to the main principles of the circular economy. The circular economy has been, in fact, created with the aim to provide a valid alternative to the dominant economic development model of linear economy. The negative effects of the linear economy, such as huge amounts of waste, high atmospheric concentrations of greenhouse gases and scarcity of resources, are indeed becoming a threat for the stability of economies [29]. According to the Ellen MacArthur Foundation [30], the circular economy is “an industrial system that is restorative or regenerative by intention and design. It replaces the ‘end-of-life’ concept with restoration, shifts towards the use of renewable energy, eliminates the use of toxic chemicals, which impair reuse, and aims for the elimination of waste through the superior design of materials, products, systems and, within this, business models” [31]. Several scientific researchers have, therefore, moved towards the development of natural or recyclable materials for FFF techniques. The development of sustainable materials for Fused Filament Fabrication, may allow a reduction in environmental impacts, indicating this technology as a sustainable manufacturing method. Several FFF filaments based on recycled materials have been recently proposed and fully characterized. The development and use of green or recycled FFF filaments has caused a series of advantages, such as a reduction in disposal and production costs, a greater availability of raw materials, a lower environmental impact, better performance, and versatility in different application sectors (engineering, medical, industrial, architectural...). However, in the field of Cultural Heritage many steps forward still need to be taken. The aim of this review is to provide a detailed overview of the recent innovations on the developing of polymer-based innovative green FFF filaments. The relationship between process, structure, and properties of the sustainable material for FFF, related to the different possible applications filed, is widely reported. Finally, the possibility to use FFF techniques in the field of Cultural Heritage restoration is extensively analyzed.

## 2. Fused Filament Fabrication (FFF)

Additive manufacturing (AM) techniques produce 3D components starting from a computer aided design (CAD) model of the part, by using a layer-by-layer construction method [32]. The first AM technique was stereolithography (SLA), introduced in the mid-1980s, in order to produce prototypes, generally characterized by significantly inferior mechanical properties when compared with parts made using other traditional manufacturing methods. Stereolithography is an additive manufacturing process that, generally, works by focusing an ultraviolet (UV) laser on to a vat of photopolymerizable to draw a pre-programmed model on to the surface of the photopolymer vat. When the UV laser touches the photopolymerizable resin, a photopolymerization reaction starts, converting the liquid photopolymer to a solid layer resin [33]. Later, the build platform reduces one layer, and a blade recoats the top of the tank with other photo-resin. This process is repeated for each layer of the design until the 3D object is complete. Completed parts must be washed with a solvent to clean wet resin from their surfaces [34].

**Figure 1:**
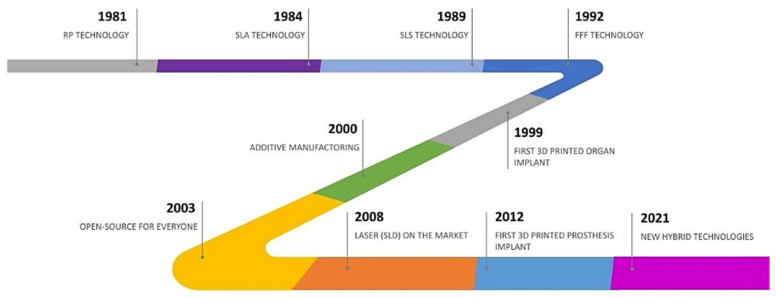
Time evolution of the AM machines.

Recently, a different method for the photopolymerization of the SLA resins, was implemented in so called “inverted stereolithography”. This allows us to print objects “bottom up” by using a vat with a transparent bottom and focusing on the UV or deep-blue polymerization laser upward through the bottom of the vat [35]. An inverted stereolithography machine starts a print by lowering the build platform to touch the bottom of the resin-filled vat, then moving upward the height of one layer. The UV laser then writes the bottom-most layer of the desired part through the transparent vat bottom. Then the vat is “rocked”, flexing and peeling the bottom of the vat away from the hardened photopolymer; the hardened material detaches from the bottom of the vat and stays attached to the rising build platform, and new liquid photopolymer flows in from the edges of the partially built part. The UV laser then writes the second-from-bottom layer and repeats the process. An advantage of this bottom-up mode is that the build volume can be much bigger than the vat itself, and only enough photopolymer is needed to keep the bottom of the build vat continuously full of photopolymer. This approach is typical of desktop SLA printers, while the right-side-up approach is more common in industrial systems [36]. Stereolithography needs the use of supporting structures which connect to the elevator platform to avoid deflection due to gravity, resist lateral pressure from the resin-filled blade, or retain newly created sections during the “vat rocking” of bottom up printing. The supports must be removed manually after printing [36]. Other forms of stereolithography build each layer by LCD masking or using a DLP projector [37]. In the 1980s, the fused deposition modeling (FDM) technique was proposed, for the first time, and commercialized by Stratasys Inc., USA, in the early 1990s. Starting from these two techniques, several machines based on the same principle of a layer-by-layer construction have been developed. A schematic time evolution of the AM machines is reported in Figure 1. 

**Figure 2:**
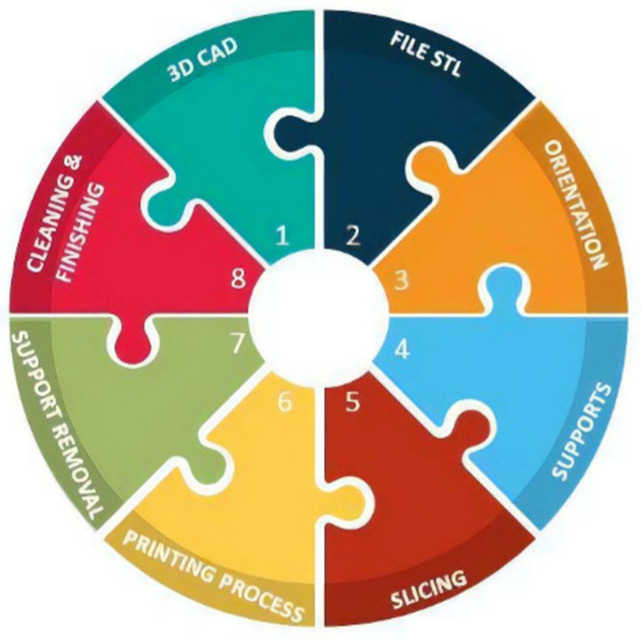
AM general procedure.

Each AM technique allows the creation of the virtual solid model, by breaking down this model data into a series of two-dimensional (2D) cross-sections and transferring these broken data to AM machine, in order to produce the physical part, layer by layer [38]. The general procedure to produce a component by using AM techniques is reported in Figure 2.

**Figure 3:**
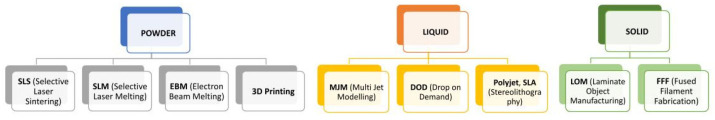
Classification of AM techniques.

From their origins, AM technologies have been used for creating models and prototypes (Rapid Prototyping), end-use parts (Rapid Manufacturing), and long-term tools for mass production of parts (Rapid Tooling) [39,40,41]. AM techniques are largely classified as (from ISO/ASTM standard 52900:2015) [42]: vat polymerization (SLA); material jetting (Objet); binder jetting (3DP); material extrusion (ME/FDM); sheet lamination (LOM); powder bed fusion (SLM/SLS); and directed energy deposition (LENS). These systems vary in terms of maximum space required, cost, building layers and type of materials used [43]. A possible classification based on the material used is reported in Figure 3.

This review is particularly focused on the Fused Filament Fabrication technique (FFF), which is fully described below.

**Figure 4:**
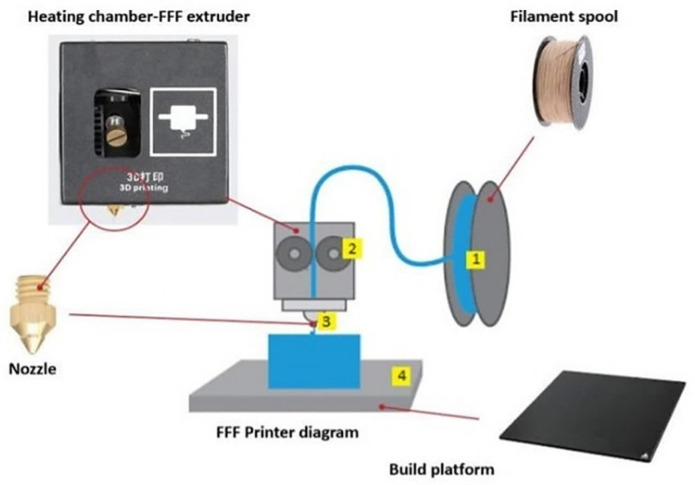
A schematic illustration of the Fused Filament Fabrication (FFF) process.

In the Fused Filament Fabrication (FFF) method, a three-dimensional (3D) geometric model is manufactured, starting from a digital project. As illustrated in Figure 4, the FFF process usually consists of the pressurization and melting of a polymer thermoplastic filament in a liquid and its subsequent deposition on the building platform through with a nozzle. 

In detail, a digital (CAD) model is transformed into a machine-readable format such as stereolithographic (STL) and additive manufacturing format (AMF) for creating components by the AM process. During the next step, the 3D model is sliced into multiple layers. The 3D model is then built by depositing layer upon layer. A programming language (G-code) is used to control the movement of the FFF extruder in the XY plane of the machine. Most of the commercial FFF machines use specific software for slicing and generating G-code. However, sometimes, the STL file is directly uploaded to the FFF machine software. The values process parameters (print speed, build orientation, and infill density) are then specified, during the next step, generally called machine setup. In most FFF machines, during the printing step, the extruder moves in a horizontal plane, deposing the layer, by following the previously created tool path. Once a layer is deposited, the build platform moves downward in the z-direction. The next layer is deposited over the last layers, and it repeats until the production of the model is finished. The strength of the built part depends on the bonding between two consecutive layers. Adequate heat energy is necessary to activate the surface of the former deposited layer and allow adhesion between the activated surface and the newly deposited layer. The final properties of the build models are strongly influenced by the FFF process parameters (surface roughness, dimensional accuracy, and mechanical properties) [44]. A list of FFF process parameters and the main final properties of the printed objects, as a function of the used materials is shown in Table 1. 

This paper does not include a detailed description of FFF process parameters, as it aims to analyze FFF filament materials, focusing, on recycled materials, with a special focus on Cultural Heritage applications [10]. However, before examining filament materials, a brief description of the relationship between the FFF process parameters and the quality of the printed model is now reported.

It is well known in the literature that FFF process parameters can affect the final properties of the printed models, such as tensile strength, compressive strength, flexural strength, dimensional accuracy, surface finish, hardness, yield strength, and ductility. Important aspects for both manufacturers and customers, i.e., the production time and costs, are also influenced by the FFF process parameters. Recently, vast experimental activity has been performed with the aim to optimize FFF process parameters, in order to increase mechanical and thermal properties, surface roughness and to decrease material wastage and building time. However, it is still necessary to deepen this aspect in order to improve the quality of printed parts. The quality of the parts made through FFF techniques, in terms of strength and accuracy are, in fact, still lower than to those possessed by the same parts made from standard processes, such as injection molding. On the other hand, the traditional manufacturing techniques do not allow to easily produce complex shapes, as it is certainly possible, by using FFF machines. For this reason, it is important to analyze the relationship between process parameters and the final properties of the FFF printed parts. Most of the published papers study the process parameters of the FFF process for FFF commercial filaments, such as ABS, PLA and PC. However, there is a gap related to the relationship between printing parameters and final properties of non-conventional materials, such as composites, nanocomposites, or recycled materials. Recently, due to the increased competition and technological advancements, some researchers are working on developing new materials, with increased final properties, for different applications such as tissue engineering, automotive, aerospace, and cultural heritage. However, enough research activity has still not been performed on the relationship between process parameters and final properties of the new materials for FFF machines. It would be important, for example, to increase the range of melting temperature of the materials used in FFF, to limit the negative influence of the humidity towards the standard FFF filaments and to develop different combinations of natural fibers and polymers, which are environmental friendly [43]. 

## 3. Materials for FFF Technology 

**Figure 5:**
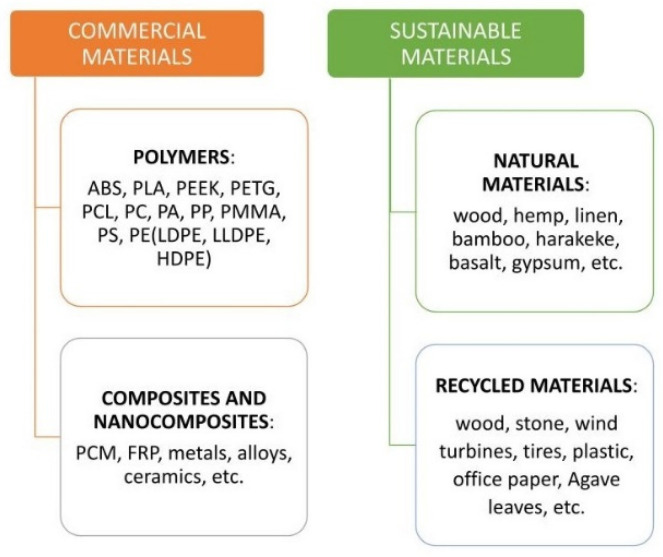
Summary diagram of the materials used in the FFF technology, reported in the following paragraphs.

Several studies have recently been performed to increase the range of materials available for FFF printing, involving the optimization of process, of printing parameters and the use of different and sometimes uncommon materials. This has led to the growth of the utilization of the FFF methodology in various manufacturing sectors. In this paragraph, the materials used in the FFF technique are reported (Figure 5), dividing them into commonly used materials, such as polymers and composites (even nanocomposites), and into “sustainable materials”, so named by the authors and divided into natural and recycled materials. In fact, the article intends to focus attention on the development of sustainable materials for FFF printing, through a general overview of the scientific research conducted in recent years on biodegradable/green or recyclable materials, highlighting once again the importance of this technology in reducing the environmental impact.

### 3.1. Commercial Materials

#### 3.1.1. Polymers

In commercially available FFF technologies, the heating element of the machine, usually, has a maximum operating temperature of about 300 °C; this implies that materials with a low melting point can be easily used with this technology [43]. Thermoplastic polymers are the materials that are mostly used in 3D printing, thanks to the workability, adaptability to the printing process, diversity, and multiplicity of shapes available on the market. They also provide sufficient strength to the final objects, giving them versatility, too [43]. The scientific literature reports a mechanical resistance, in terms of tensile strength, between 1.5 MPa and 150 MPa [41,45,48,49,53,57].

**Figure 6:**
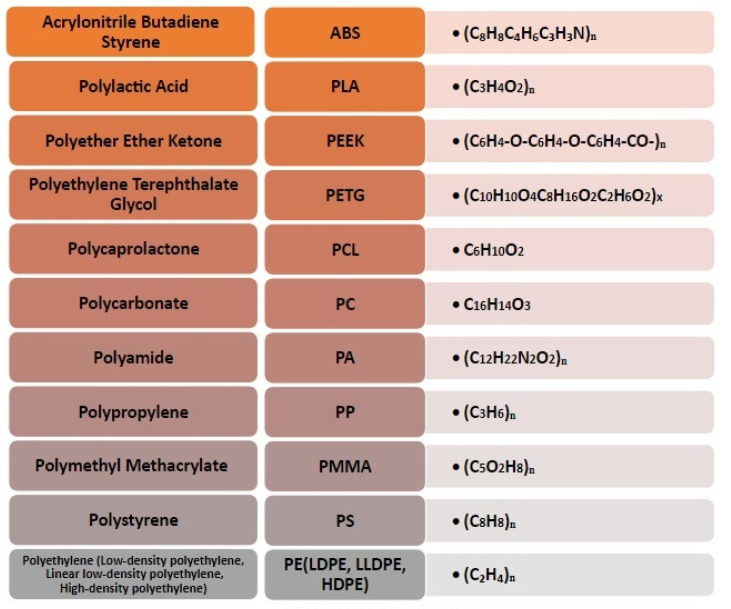
Common polymers used for FFF technology and chemical formula.

The most used thermoplastic filaments for FFF process are reported with the respective chemical formula in Figure 6.

The most common and commercially available FFF filaments are the acrylonitrile butadiene styrene (ABS) and the polylactic acid (PLA) [45,58]. ABS and PLA possess thermal (melting point, glass transition temperature, etc.) and rheological properties, that can be easily processed using FFF technology [43]. 

ABS is an amorphous and thermoplastic polymer made from petroleum. It is not biodegradable, and it is extruded at high temperatures (around 220–280 °C). The literature reports the following mechanical properties for ABS: tensile strength between 13.0 to 65.0 MPa, Young’s modulus between 1.00 to 2.65 GPa, and flexural strength equal to 66 MPa [45,46,49,57]. ABS is widely used in industry, due to its impact resistance and toughness, for example for prototyping, production of toys and components for boats and cars.

A recent manuscript of Algarni and Ghazali (2021) has also shown that some process parameters (raster angle, layer thickness, infill percentage and printing speed) of FFF methodology, can influence the mechanical properties of the polymers. In the case of ABS, the infill percentage seems to be the parameter that most affects the properties of ABS printed models [45]. However, besides mechanical properties, other material characteristics must be considered to select the most appropriate polymer for the FFF process, such as biodegradability, a-toxicity, reproducibility, low cost and availability [41,43]. Unfortunately, ABS is a non-biodegradable material and it has a medium toxicity [43]. 

Polylactid acid (PLA) is the other thermoplastic polymer, commonly used in 3D printing, by FFF technique. It is a bio-based, biodegradable and biocompatible polymer [58]. The extrusion temperature varies from 160 to 230 °C [45,57]; it has a tensile strength between 35 to 65 MPa, Young’s modulus equal to 2.3 GPa, and a flexural strength of about 97 MPa [45,46,49,57]. However, its most important disadvantage is the great sensitivity to high temperature (about 200 °C), which induces the degradation of the macromolecular structure. The printing parameter, which positively influences the mechanical properties of PLA 3D printed models, seems to be the infill percentage, as reported by Algarni and Ghazali in 2021 [45]. However, Cao and Xie (2017) show a greater influence of the raster angle on the Young’s modulus of the PLA parts, obtained by FFF, too [59].

Other polymers that can be used for 3D printing, are polyetheretherketone (PEEK) and polyethylene terephthalate glycol (PETG). The first is a thermoplastic biomaterial with good thermal resistance and stability, and excellent mechanical properties. Its extrusion temperature varies from 340 to 440 °C [45,51]; it has a tensile strength equal to 100 MPa and Flexural strength equal to 170 MPa [45,51]. It is used for the manufacture of aerospace components, as well as for medical support for the regeneration of human bone tissues [46,48,51] The major limit of the PEEK is the non-biodegradability. Algarni and Ghazali in 2021 have demonstrated that the Young’s modulus and the ultimate tensile strength of PEEK are mainly influenced by the infill percentage. The flexural strength and the fractural strain were significantly affected by the printing speed and by the infill percentage, respectively [45].

PETG is a thermoplastic polymer that derives from the polyethylene terephthalate family. It is particularly durable and more flexible and softer than PLA and ABS polymers. The extrusion temperature varies from 220 to 250 °C [52,57]. PETG has a tensile strength equal to 49 MPa and a flexural strength equal to 70 MPa [52,57]. PETG is widely used in implant medicine and in food packaging. The infill percentage increases the Young’s modulus of the PETG, the printing speed affects the flexural strength, while the raster angle affects the elongation at break [45].

Other polymeric materials used in FFF technology are polycaprolactone (PCL), polycarbonate (PC), polyamide (PA) or nylon, polypropylene (PP), polymethyl methylacrylate (PMMA), polystyrene (PS), polyetherimide (PEI), and various types of polyethylene (PE), including low density polyethylene (LDPE), linear low density polyethylene (LLDPE), high density polyethylene (HDPE), and polyethylene terephthalate (PET) [5,32,45,49,52,55,56,57,60,61,62]. A combination of the polymeric materials has been used in some scientific publications, such as ABS and PC, PLA and PC, PE and PP, and so on [32,60,62].

These materials are commonly used to print automotive components, surgical and medical objects, prototypes, toys and many other everyday products [41,50,63]. The thermal and mechanical properties of the previously cited polymers for FFF technique have already been summarized in Table 1. Although thermoplastic polymers of Table 1 are commonly used in 3D printing extrusion technologies, most of these materials are not eco-friendly. The degradation times are often long and depend on the different environmental conditions [36]. In addition, their final properties could not be suitable for structural applications. In the next sections the possibility to improve both the mechanical properties and the sustainability of the 3D printable materials is discussed.

#### 3.1.2. Composites and Nanocomposites

The need to have materials with advanced performance has led to the production of polymer matrix composites (PMC) and nano-composites for FFF process (Table 2).

In detail, different kinds of reinforcement materials have been added to the polymeric matrix in order to improve the properties of the neat thermoplastic polymer. In this way, the final properties of the 3D printed objects, such as adhesiveness, flexibility, conductivity, process capacity, toughness and resistance depend on that of both materials, and, in particular, on the composition of the matrix and the type of reinforcement materials used [43,79,80]. For example, metal powders are often used as reinforcing materials [72,79,81]. Aluminum and iron powders are the most commonly used fillers for PMC, that have ABS, PP and PA as a matrix [69,72,82]. These PMC show improved mechanical performances, although they have a decreased viscosity due to the presence of the metal powder. This can represent a limit, which can be solved, by using surfactants and plasticizers [43,83]. The thermal properties of the PMC/metal powder, also, increase with the diameter of particle size of the filler [69]. However, the data available in the literature on the evaluation of electrical and magnetic properties are still few.

Ceramic materials are another type of filler added to polymeric matrices, especially useful in biomedical applications and tissue engineering, where biocompatibility is required [15,17,82]. These materials are called polymer ceramic composites or biocomposites, and calcium ceramics, TiO_2_, ZrO_2_ and Al_2_O_3_ are mostly used as reinforcement materials with polymer matrices such as PLA, PA, PP, PCL, PEEK, PMMA [60,66,68].

Metals and ceramics are also used in the form of nanoparticles, to improve the mechanical and thermal properties of polymeric composites: these materials are called nanocomposites and exhibit excellent thermal, mechanical and transport properties [15,16,17,81,84,85,86]. Specifically, these nanoparticles include exfoliated clay and exfoliated graphite, carbon nanotubes, carbon nanofibers, nanocrystalline metals and a range of other nano-sized inorganic fillers [41,43,84]. Three different dispersion states of inclusions occur when nanoparticles are added to the polymer matrix, such as immiscible, intercalated and exfoliated [84]. Among these, exfoliation is often indicated to improve miscibility and mechanical properties of the filament for FFF, especially in the oriented direction of the filament. In the perpendicular direction of the filament, it is more difficult to have a significant improvement of the mechanical properties with the addition of nanoparticles, due to the interfacial bonding between two adjacent printed layers, which plays a significant role [84]. Many works have been reported to improve the final performance of nano-composite filaments and 3D printed objects [60,76,77,84]. For example, Cobos et al. (2020) added melanized linseed oil (MLO) to the nanoparticles as a lubricant, and studied its influence on the thermal and rheological properties of polylactic acid/multi-walled carbon nanotubes (PLA/MWCNTs) and halloysite nanotubes nanocomposites (PLA/HNT), obtaining an increase in the fluidity index of 46% [87].

Nanoparticles are also widely used in thermal and conductivity applications. The addition of highly conductive fillers, such as graphene oxide, graphene nanoplatelets [66], graphite nanopatterns (GNP), boron nitride (BN), carbon nanotubes (CNT), MWCNT, metals and others allow the limits due to the low thermal conductivity (TC) of the polymer matrix (generally 0.1–0.5 W m^−1^ K^−1^) to be overcome [88]. Silva et al. (2021) have developed tissue-engineered composite filaments of PLA reinforced with graphite nanoparticles (PLA + EG), chemically functionalized (PLA + f-EG), or functionalized and decorated with silver nanoparticles (PLA + ((f-EG) + Ag)) [86]. The composite filaments produced are thermally stable. Furthermore, the incorporation of graphite increases the stiffness of the composites and decreases the electrical resistivity, compared to the electrical resistivity of PLA [86]. Nanoinclusions have also been used to improve antibacterial and biocompatible performance in tissue engineering [84,86]. Bayraktar et al. (2019) developed 3D printable antibacterial composites by adding silver nanowires (AgNW) in small percentages to the PLA matrix [78]. The AgNWs showed excellent dispersion within the matrix. Furthermore, the antibacterial properties of AgNW/PLA nanocomposites were effective against both Staphylococcus aureus (S. aureus) and Escherichia coli (E. coli) [78].

Ceramic nanoparticles are also suitable for tissue engineering applications, because they promote cell growth and improve the bioactivity of the bone implants used. For example, Esposito Corcione et al. developed composite filaments for FFF with PLA polymer matrix and hydroxyapatite (HA) filler [15,16,17]. Different percentages of HA were added to the polymer (from 5 to 50% HA), using an on-step solvent-free process. In addition, the adaptability of the filament PLA/HA to the printing process was tested using a 30% HA content [17]. Other types of nanoparticles have been added to polymer matrices to modify the performance of composite filaments. For example, nanoclay is one of the most studied nanoparticles for the production of composites. It is a natural mineral belonging to the smectite family, of which montmorillonite (MMT) is perhaps the most investigated [89]. Polymer-nanoclay nanocomposites possess better properties than pure polymers, such as higher strength, modulus and heat deflection temperature [89]. Other nanoparticles are, for example, silica, which have given greater thermoelasticity, increased handling and performance quality of thermoplastic polymers, or polyhedral oligomeric silsesquioxane (POSS) nanoparticles, that have increased flexural strength (22%), flexural modulus (9%) and toughness (117%), compared to pure PLA [85]. Furthermore, Pezzana et al. 2021 demonstrated the possibility of increasing the thermal conductivity and thermal conductivity of a silicon-acrylate nanocomposite using boron nitride (BN) nanoparticles [90].

Some polymeric composites also include the use of fibers as reinforcement, usually glass or carbon fibers [48,74,75,89]. The fiber-reinforced plastics (FRP) composite, widely used in aerospace and automobile industrial sectors, was born to increase the efficiency and performance of the vehicle, using lighter materials as substitutes for some heavy components [48,63,91]. Three different processes of incorporating the fibers into the polymer matrix are usually employed in the FFF technology. The first involves the realization of the fiber-matrix filament to be extruded in the 3D printer. In the second method, the fibers and the matrix are initially separated and mixed directly in the print head, giving the possibility to modify the quantities of each, directly in this phase. In the last, more versatile method, the fibers are directly incorporated into the polymer component, using a separate mechanism [48]. Pervaiz et al. (2021) wrote a review of the fiber-reinforced matrix composites used in FFF technology, analyzing the existing scientific literature on this issue. In the work, they report the materials usually used as a matrix and their properties, the reinforcing fibers usually used and the fields of applications, the physical and mechanical characteristics, the variations of their properties depending on the type, quantity, printing parameters, advantages and disadvantages etc. [48]. Many works deal with the addition of fibers to thermoplastic polymers, but recent studies are also looking at the use of thermoset-based composites. For example, Mantelli et al. (2021) used the UV-assisted 3D printing process to produce thermoset composites reinforced with carbon fibers (CFs) [92]. The addition of CFs in the matrix led to a significant increase in toughness and elastic modulus. However, further studies are investigating the use of a sizing agent to expand the performance of these thermoset-based composites [92]. Recent research also reports the use of innovative materials as reinforcement of the polymer matrix. For example, Loh et al. (2021) have developed and tested a polymer-textile composite [93]. The authors used PLA as a polymer for the direct extrusion of the material (ME), three different combinations of synthetic textiles and polymers (two based on PLA and Nylon with different mesh, one based on PLA and Polyester), using the Fused Filament Fabrication technology for manufacturing polymer–textile composites. The aim of the work was to investigate the effects of varying textile substrate parameters, such as types of fibers, fabric weight, weave pattern, weft density and surface properties on the polymer–textile adhesion force, while also optimizing the printing parameters and evaluating the best mechanical performances. Better behavior of Nylon is highlighted compared to the other fabrics studied, from the tensile tests reported in the work (the initial force exceeded 40 N, maximum extension of about 20 mm compared to Polyester). The compatibility between the printing material and the type of fiber has a dominant effect on the resistance of the polymer-textile composites. The authors also highlight the importance of AM technologies for the development of innovative and sustainable models for the textile industry [93].

Overall, the limits of the composite materials illustrated in this paragraph are for example the non-biodegradability, the waste disposal, the costs related to this process and the significant impact on the environment. Scientific researchers have therefore moved towards the development of natural or recyclable materials, as detailed in the following paragraphs. 

### 3.2. Sustainable Materials

#### 3.2.1. Natural Materials

Recent researches have involved the use of natural materials for the production of composite filaments, obtaining various advantages, such as low cost, low density, availability, biodegradability [46,49,94]. The development of sustainable materials for 3D printing, in particular for Fused Filament Fabrication, may allow a decrease in environmental impacts, demonstrating the importance of this technology as a sustainable method of manufacturing. Various filaments for FFF printing have been developed, using natural materials as fillers, by academic researchers, companies, start-ups or within international projects (Table 3). 

Many of these include, as a filler, wood of different plant species, different particle sizes and different binders. In 2021, Das et al. wrote a review on the use of wood dust for 3D printing, summarizing the final properties the 3D printed products and their potential future applications [94]. The wood filaments offer some advantages, such as biodegradability, non-toxicity, low deformation, and good elasticity. On the other hand, they have limits due to the rigidity provided by the wood fibers and due to required management conditions, as they must be stored in a cool and dry place, with a temperature ranging between 15 and 25 °C, away from UV rays and heat sources [94]. Wood biocomposites for 3D printing usually show high porosity, a lack of adhesion with the matrix, swelling and greater water absorption, as well as a decrease in mechanical properties with an increase in the filler content. However, several studies have been published, with the aim of optimizing the performance of the filaments, working on the quantities of fillers, on the possibility of adding additives and binders, and on the printing parameters, such as the extrusion temperature [46,94]. Gkartzou et al. (2017) studied the addition of low-cost kraft lignin to PLA to produce a filament, using Fused Filament Fabrication (FFF) 3D printing process [95]. The morphological, mechanical and thermal properties of the biofilament, at different lignin concentrations (from 5 to 15% by weight), were studied. This study has shown that the fragility of PLA increases with the increase in lignin: a significant reduction in the region of plastic deformation of the stress-strain curves and a disappearance of the yield point is observed. SEM images showed an increase in the surface roughness of the PLA/lignin filament, in addition to the formation of aggregates. However, lignin has no negative effect on Young’s modulus of elasticity. A lignin content equal to 5% by weight was finally selected for the production of filaments for 3D printing (diameter of 1.78 ± 0.04 mm). Various extrusion variables were evaluated, such as temperature and printing speed, selecting the following values as optimal: optimal extrusion temperature equal to 205 °C, printing speed 20 mm/s [95]. In the same year, Tao et al. (2017) developed a composite filament for FFF, consisting of poplar wood flour (WF, 5% by weight) in a PLA matrix, achieving the following results: the addition of WF modifies the surface microstructure, but improves the deformation resistance of the composite and there is no effect on the melting temperature [102]. Daver et al. (2018) developed a PLA and cork biofilament with the addition of Tributyl citrate (TBC) as a plasticizer, to overcome the brittleness of PLA [96]. Kariz et al. (2018) in their papers used dust from beech wood, especially *Fagus sylvatica* L. [47,97]. Six filaments using polylactic acid (PLA) with varying loading levels of wood particles from 0% to 50% by weight were produced. The study of the morphological and mechanical properties showed a decrease in density and an increase in the roughness of the filament as the percentage of wood increases, which causes the formation of agglomerates up to the occlusion of the printing nozzle. The tensile strength of the filaments after a slight initial increase (from 55 MPa to 57 MPa with an addition of 10% wood), decreases with higher levels of wood (30 MPa for filaments with 50% wood content) [47]. The same authors studied the effect of humidity on these samples, exposing the 3D printed samples containing different percentages by weight of wood dust (from 10 to 50%) at different levels of relative humidity (RH): 33%, 65% and 87% [97]. The results showed that the samples made with filaments with a higher wood content had a higher moisture content, a greater dimensional swelling and a lower modulus of elasticity (MOE) [97]. In the work of Le Guen et al. (2019) pine wood (Pinus radiata species), and rice husk powder together with PLA were tested to produce filaments for FFF printing [98]. The data relating to the morphological, mechanical and chemical characterization showed colorimetric variations and a significant difference in the rheological behavior, while the mechanical properties of the 3D printed samples were found to be similar and predominantly influenced by the printing direction [98]. Zhao et al. (2016) developed a composite filament made of bamboo powder and polylactic acid (PLA), optimizing the relationship between bamboo and plastic, the amount of additives added (polyethylene glycol) and the parameters related to extrusion [99]. Despite the difficulties deriving from the environmental conditions on the process, the production process of the biofilament is not complex and the performances achieved make it suitable for industrial applications, since a good print quality has been achieved [99]. Depuydt et al. (2018) instead studied a filament based on PLA reinforced with bamboo and flax fibers, together with the addition of two plasticizers, for fused deposition modeling [103]. The poplar wood flour was instead used in the paper of Bi et al. (2018), using thermoplastic polyurethane (TPU) as a binder to make the biocomposite, whose properties have been improved by the addition of some modifiers: the diphenylmethyl propane diisocyanate (MDI) and EPDM-g-MAH [104]. The use of lignin nanoparticles (nanolignin, NL) prepared by ultrasonic treatment of kraft lignin, to obtain water-lignin dispersions with excellent colloidal stability, was investigated by Gonzales et al. (2017) [105]. NL particles were incorporated into a water-based thermoplastic polyurethane matrix at different concentrations to produce bio-based materials nanocomposites, achieving excellent performance with an environmentally friendly approach [105].

Different types of binding agents for wood to print objects are reported in the scientific literature, such as polylactic acid (PLA), thermoplastic polyurethane (TPU), urea formaldehyde (UF), polyhydroxyalkanoates (PHA), gypsum, polyvinyl alcohol (PVA) etc. [47,95,96,98,99,102,103,104,105,106]. For example, Henke and Treml (2013) used various binders with spruce shavings (sized between 0.8 and 2 mm), such as gypsum, methylcellulose, sodium silicate and cement. The 3D printing process was achieved by depositing the dry mixture followed subsequently by adding water, which acts as an activator to solidify the material [107].

Nowadays, there are many companies that have commercialized FFF wood filaments, for example the Emerging Objects Company (USA) has experimented different innovative and eco-sustainable materials in addition to wood; the ColorFabb (NL) company supplies pine/PLA/PHA wood filaments called WoodFill; the Formfutura company (NL) sells several wood filaments based on 30–40% pine (EasyWood™Pine), on cedar or coconut (EasyWOOD™ CEDAR), on birch (BIRCH Eastwood™) at 40% together with PLA and additives; the PrimaSelect™ company (SE) has marketed a filament called PrimaSELECT™WOOD, characterized by PLA and a generous percentage of wood fibers (about 35–40%), offering a wide choice on the market. Then, depending on the brand, there are different types of wood filaments, such as bamboo, birch, cedar, cork, ebony, olive, pine and even coconut!

Other natural materials have been used for the production of “green” filaments for 3D printing, in addition to wood and lignin. Yaguchi et al. (2020) have produced a biofilament with a PLA matrix and hemp fibers as reinforcement and they evaluated the durability and biodegradability of the composite by accelerated aging tests and a study of mechanical properties [107]. The results showed good biodegradability and greater strength than commercial neat PLA filaments [107]. Stoof et al. (2017) have produced filaments of both hemp and harakeke (Phormium tenax) in varying weight percentages within polylactic acid (PLA) polymer, and they used them to print tensile test samples [100]. Test results showed that composite filaments have better mechanical properties than PLA samples [100]. Sia et al. (2014) investigated the toughness of the mode II interfacial fracture of a PLA matrix composite and oil palm empty fruit bunch (OPEFB) fibers, following a surface treatment with a NaOH solution, to improve the bonding interface of the composite [108].

A review on the composites with oil palm fiber (OPF) was published in 2011, which reports the mechanical, tensile and morphological properties of composites, with different polymeric matrix (PLA, PP etc.) [109]. Osman and Atia (2018) studied an ABS-rice straw composite feedstock filament for FFF [101]. A variable fiber content (5–15%) was tested and a single screw extruder was used for the production of the filament. Finally, specimens were printed and subjected to mechanical tests, according to the ASTM standards. The results showed a decrease in tensile properties with increasing RS content and a different behavior depending on the raster angle, indicating that the tensile properties of the FDM parts are anisotropic, the flexural properties decreased with the fiber content and the water absorption of the composite increased with increasing fiber content [101]. The use of cellulose derivatives is also present in the scientific literature; for example, Tenhunen et al. (2018) investigated the use of two acetylated cellulose derivatives to modify and functionalize fabrics, through the use of 3D printing: rigid cellulose acetate (CA) and flexible acetoxypropyl cellulose (APC), together with solvents acid acetic and acetone, respectively [110]. The APC having a more branched structure was found to be less suitable for its adhesive properties, compared to the CA. According to the authors, both materials open new development perspectives for the personalization of cellulosic fabrics, without a large manpower being required [110].

Additional natural materials are currently used on the market for the production of FFF filaments. For example, the Emerging Objects company (USA, already mentioned in this work) advertises 3D products in natural materials such as tea leaves, salt and chocolate, highlighting an ever-increasing attention in the market on the issue of sustainability.

Other natural materials have recently been employed in AM methodologies. Yu et al. (2021) dealt with the production of polylactic acid composites reinforced with natural basalt fibers (BFs) (containing minerals such as plagioclase, olivine, pyroxene), similar to glass fiber and with better thermal and mechanical properties, but which are also eco-friendly [111]. PLA specimens containing 8% by volume of basalt fibers with different orientations were printed (nozzle temperature equal to 220 °C and printing speed equal to 50mm/s) and subsequently tested by High-resolution 3D X-ray microscopy (X-ray uTC image) for the analysis of internal morphology and anisotropy [111].

Similar reinforced polymeric products exist on the market, for example the KLONER3D^®^ company (IT) sells a filament for FFF in gypsum and harmless co-polyesters called LAYBRICK, which has the appearance of a stone; the Emerging Objects company (USA) prints 3D objects in sand, stone and cement, or the FormFutura company (NE) has commercialized the filament based on PLA and stone (50%), called STONEFIL, which simulates materials such as stone, clay, terracotta, granite, as well as many other companies. 

#### 3.2.2. Recycled Materials

The term “sustainability” includes the creation of products that maximize their economic and social impact and minimize harmful effects on the environment. Thus, Fused Filament Fabrication strategies for sustainability must consider not only the use of ecological/green materials, but also the use of recycled materials (Table 4). These products usually have a longer life and a reduced impact on the environment. According to Medellin-Castillo and Zaragoza-Siqueiros (2019), additive manufacturing technologies in general can give life to products that satisfy three parameters, such as economy, environment and society [41]; they also claim that an ideal product is one that maximizes all three areas, because it is good for the environment, it is profitable for the company, and it improves society [41].

As reported in the previous paragraphs of this review, the 3D printing market is a rapidly growing sector and the filaments used in 3D printing can be made with a wide variety of thermoplastic materials, including those made of recycled materials. The scientific literature reports various works related to the recycling of polymeric materials [49,50,51]. These materials find application in many areas of daily life and the topic of their recycling has become of considerable interest in recent years. After their use, plastics become persistent and harmful waste [50].

Several international projects were recently created involving the recycling of polymeric materials and 3D printing, and involving citizens in their activities. In the project called “Print your city”, born from a collaboration between Greece (Thessaloniki) and Holland (Amsterdam), plastic waste is transformed into street furniture, thanks to 3D printing [https://www.printyour.city/new-page, accessed on 30 November 2021]. The citizens of Thessaloniki (Greece) bring the bottles and plastic packaging to the “Zero waste lab”, where they can design their own customized street furniture and indicate the position in which to place them. In this way, properly treated waste is 3D printed taking new life directly on site, in front of the eyes of those who designed it. New sustainable products are created, such as benches, bike racks, exercise equipment, tree pots, dog bowls or mini-bookshelves. 

The project named “RE.CO.RD-REcycling strategies for the COastal sustainable waste management towards R&D Innovation”, born from the collaboration between Italy and Greece and financed under the Interreg VA Greece-Italy 2014–2020 Program [https://www.interreg-record.eu, accessed on 16 January 2022] concerned the experimentation of new solutions for the recycling of plastic waste produced on the coast, due to the high presence of tourist and economic activities. The project activities concerned the recycling of plastics through the use of 3D printing and the transformation of waste into new products, with the ultimate aim of reducing the environmental impact and pollution of the sea. As part of this project, a manuscript published by Ferrari et al. (2021) concerns the production of filaments from PET bottles collected by the sea [112]. The collected bottles were suitably treated and after optimizing the extrusion parameters, the filament was used to produce 3D printed samples using the FFF technique. All the produced samples were tested and compared with those obtained using commercial PET pellets, in order to compare the effect of aging on thermal, morphological, and mechanical properties. The results showed a progressive decrease in the degradation temperature of the recycled PET with increasing processing cycles. The tensile tests showed the difference in mechanical response due to the predominance of the crystalline or amorphous phase in the sample, but overall good mechanical behavior was found for the 3D printed samples [112]. Farina et al. (2019) studied the development of a filament for FFF starting from recycled Nylon-6, combined with ABS and TiO_2_ [113]. The study of rheological, thermal and mechanical properties shows the following parameters: tensile strength at yield in the range of 76.20–86.91 MPa, Young’s modulus of 1.64–2.34 GPa and a wear resistance of 92 m [113].

Wei et al. (2021) dealt with the production of a filament starting from the recycling of aluminum-plastic packaging, a multilayer material composed of polymer and aluminum (APWW) [114]. Following the solid state cutting milling (S3M) and the manufacturing of the filament by combining the powder of APPW/expandable and graphite composite (EG) in percentages of 10% or 20% EG, the extrusion process was started by FFF method. The study of the morphological, mechanical and thermal properties conducted by the authors has shown that the tensile strength is 13.58 MPa, the thermal conductivity of the filament is equal to 2.7 W/mK, higher than the pure APPW filament. The mechanical properties are also excellent, and the filament is suitable for 3D printing and thermal applications [114]. Mikula et al. (2021) have instead produced an interesting review on the recycling of polymeric materials for the production of filaments for 3D printing [58]. The problem of polymeric materials as waste is greater for HDPE, LDPE, PP and PVC, plastics widely used by manufacturers and which end up in landfills with greenhouse gas emissions, while fewer problems are given by the use of biodegradable PLA. The authors report a series of works related to the recycling of PP, PS, PE, ASA, PET, PVC, ABS, HIPS, PC, TPU, HDPE, LDPE, Nylon and PLA. The recycling phases usually involve cleaning, grinding, melting, extrusion and measuring the properties of the filament, the possibility of adding binders or proceeding with solubilization in organic solvent and the use of reinforcing material/additives. The study of the final properties of the filament that undergo changes after recycling are also reported for each publication, as well as the commercial filaments made from recycled polymers and the companies that have commercialized these filaments, such as B-PET filamentive, Fila-cycle, Refil, Innofil3D etc. [58].

Cellulose and wood have been reported in the paragraph “Natural materials” as materials used for the production of filaments for FFF. Cellulose has a very low environmental impact, having a totally natural origin. Furthermore, the finished printed product is very elastic, economical and light. Nowadays, many companies have commercialized filaments with reused wood as reinforcement. Among these, there are also companies that offer filaments for FFF in cellulose and recycled wood, such as the company Giantarm (China), which supplies a filament with a percentage of 20% of recycled wood fibers; KLONER3D^®^ (Florence, Italy) has commercialized the so-called LAYWOOD filament, that containing about 40% of recycled wood in a polymer matrix, or Eumakers (Barletta, Italy) which sells the so-called PLA Woodfir, an high quality 3D printing PLA filament blended with fir-wood fibers, and others. The Kanèsis Startup, whose name comes from the fusion of hemp and kinesis (movement), has created a new bioplastic called HBP^®^, starting from vegetable waste from Hemp, which is perfectly suitable for use with FFF technology. This addition to the polymer matrix gives the printed object a much higher mechanical resistance than the same object printed with traditional polymers, for example with ABS or PLA, while maintaining a lower weight of about 30%.

Also, in the scientific literature there are manuscripts on the recycling of cellulose materials. For example, John et al. (2021) have developed a biopolymer for 3D printing applications from forestry waste residues [115]. The developed biopolymer contains polylactic acid/polybutylene succinate (PLA/PBS) and cellulose nanofibers (CNFs) derived from recycled cellulose (sawdust) and after chemically modified. The study of the mechanical and thermal properties was carried out both on the PLA/PBS mixtures and on the bio-nanocomposites PLA/PBS/CNFs containing functionalized and non-functionalized cellulose nanofibers. The best performances were obtained by adding functionalized cellulose nanofibers to the PLA/PBS matrix, because the hydrophobicity and the crystallinity of the mixtures improve, due to the nucleating effect. Finally, the authors also report an example of 3D printing of food packaging boxes using PLA/CNFs filament [115].

An interesting research by Tao et al. (2021) instead concerns the development of a composite filament for 3D printing, based on the recycling of office paper (WOP) in PLA matrix [120]. Different concentrations of WOP (5%, 10%, 15% by weight) were added and the morphology, rheology, thermal and physical properties of the filaments were investigated. The results show that the Tg and Tm of the composites remained similar to pure PLA. The tensile strength decreases as the WOP content increases. In addition, the work also demonstrates that the use of a silane coupling agent (γ-methacryloxypropyltrimethoxylsilane, KH570) to modify the WOP granules, increases the adhesion between WOP and PLA and improves the mechanical properties. Filaments made from recycled office paper show no degradation below 260 °C and it is suitable for 3D printing [120]. The paper of Velarde et al. (2021) concerns the production of filaments for FFF, based on polylactic acid (PLA) and fibers from Agave leaves, a waste material from the production of tequila [116]. The authors compared the morphological, mechanical and thermal properties of filaments made of PLA and various percentages by weight of agave leaves (3, 5 and 10%), as well as studied the adaptability of the filament to printing via FFF, using two different raster angles (−45°/45°, 0°/90°). The results reported in the paper indicate that the fiber content strongly influences the crystallinity (increases from 23.7 to 44.1%) and the porosity and the bending properties of the final biocomposites, while the raster angle influences more the morphology and the impact resistance of printed biocomposites. However, printed biocomposites appear to be suitable for the fabrication of 3D printed objects, thanks to the low cost, compostability, and low density [116].

Studies on less common recycled materials are also reported in the scientific literature. For example, Tran et al. (2017) dealt with the production of 3D printing filaments in biodegradable poly (ε-caprolactone) (PCL) and micronized powder derived from cocoa shell waste (CSW) as filler [121]. Different weight concentrations of fillers with particle diameters of about 50 microns, added to the polymeric matrix, were studied up to a weight concentration of 50%. PCL was selected by the authors for the polymer matrix instead of the usual PLA and ABS, since it is a semi-crystalline synthetic polyester with excellent properties of biocompatibility, biodegradability, flexibility, high elongation at break, workability (low melting point ≈ 60 °C and high decomposition temperature 350 °C), for which less energy required for printing, unlike PLA and ABS. The filament with a diameter of 1.75 mm was produced with a single-screw extruder, the properties (SEM, ATR-FTIR, XRD, DSC, TGA) and the adaptability to 3D printing (using FFF) were subsequently studied. The results reported by the authors indicate sufficient thermal and mechanical properties of the eco-friendly biofilament produced: the adhesion between the printed layers is adequate, as well as the resolution of the final object, so it could be used for biomedical applications or the production of everyday objects, design objects, toys, etc. [121].

Used car tires are the largest and most problematic sources of waste in the world: an enormous volume of tires are manufactured each year, but they are not biodegradable, and they contain a number of components that are ecologically problematic, such as the metals. The Emerging Objects Company (Adeline, CA, USA) has developed a formula for using recycled rubber content in 3D printing using tires that are cryogenically, reduced to a micronized rubber powder with many possible applications in the building industry: for example to stamp design object, outdoor furniture, or wall panels that can be used for acoustic and sound dampening purposes. TreeD Filaments company (IT) has developed the so-called Pneumatique, a filament based on thermoplastic elastomer and reactive rubber of recycled tires. The company said that from one single tire it is possible to obtain enough pellet for the extrusion of nine pneumatique filament spools of 500gr each, reducing pollution problems. 

The manuscript of Rahimizadeh et al. (2020) is based on the use of wind turbine waste for the production of a composite filament for 3D printing, with a polymer matrix in PLA [117]. The material used in wind turbines is mainly composed of 85% of materials such as copper and steel, and the remainder of glass fibers. Glass fibers cause more disposal problems and the authors developed this paper with the aim of developing new recycling methods. Different amounts of recycled fibers were used, such as 3, 5, and 10 wt% and the filament was tested, using thermogravimetric analysis (TGA) and micro computed tomography (μCT). The result showed an increase in the tensile strength and modulus of 20% and 28%, respectively, when a fiber content of 5% is used in the filament, compared to pure PLA samples. Moreover, the authors highlight that the use of long fibers leads to higher tensile strength, stiffness, and failure strain, compared with the short fibers. Overall, the research is presented as a sustainable solution for the reuse of glassy materials in general [117]. 

The activities of recycled materials for the production of filaments to be used in FFF methodologies also involved stone materials, used as fillers in polymeric matrices. Among the stone materials, marble is one of the most used materials in the world, especially in the civil engineering sector, the disposal of which risks becoming an environmental problem. Ledvai et al. (2021) reused the marble dust (MD), coming from the cutting of bricks, for the production of a composite filament with a polymeric matrix (PLA) by extrusion molding [118]. Specifically, an amount of MD by weight of up to 20% was used to produce the composite filament, with a particle diameter of 5–15 μm. The authors studied the mechanical, morphological, thermal properties and resistance to wear, with the ultimate aim of investigating the possibility of recycling this material, reducing business costs. The results show that the mechanical properties, both tensile and flexural modulus, improved significantly up to the 10% concentration of marble dust, while a slight loss was observed in strength and deformability. Wear resistance increases with the MD content. The maximum values are reached using 20% of MD. Overall, the composites with improved properties compared to pure PLA have been developed, and these can be used in different application areas, thanks to reduced cost [118].

Several companies and startups are dealing with the recycling of marble for prototyping applications, such as the company Marble EcoDesign (IT) which is developing a new 3D printing technique (FFF) using waste materials from common excavation operations of the marble, mixed with powders/resins catalyzed with UV radiation, with the aim of safeguarding the ecosystem of the territories, in which the mass production of marble is most developed. The innovative startup, TRIP (Techniques Recovery Innovative Printable), is also dealing with the recycling of marble processing residues to be used as raw material for a new 3D printing technique. The idea, born in 2014 and materialized in 2015, involves Inter Marmi Srl company from Trani (Puglia, Italy), highly qualified in stone processing nationally and internationally. The project is aimed at achieving a closed production cycle, in which each industrial district minimizes waste materials and the production of landfill waste, avoiding the complex and high-cost disposal process.

Corcione et al. (2018) have published a manuscript on the reuse of Lecce stone waste for industrial design and building applications, using the FFF technique [119]. Pietra leccese (PL) is an Apulian Miocene limestone widely used since ancient times by local artisans, and especially as a building and ornamental material during the Baroque period. However, it is a non-renewable resource, and its life cycle ends with a large amount of waste, both in solid and muddy form. 3D filaments were obtained with a twin-screw extruder, using polylactic acid and lecce stone waste powder in concentrations of 50 and 60% by weight. The thermal, mechanical, and rheological properties have been tested. The results showed that the degradation temperature of PLA is not much affected by the PL filler and remains almost unchanged at about 170 °C. The glass transition temperature increases slightly from 56 °C to 66 °C, while the viscosity is characterized by the pseudoplastic behavior, typical of thermoplastic polymers: it increases according to the stone content but it is comparable to that of conventionally used polymers in the FFF, in these concentrations. The adaptability of the PLA/PL composite filament to the extrusion and printing process was demonstrated by the authors, as well as the possibility of closing the stone production cycle, on behalf of a circular economy [119].

## 4. Applications: Focus on Cultural Heritage

**Figure 7:**
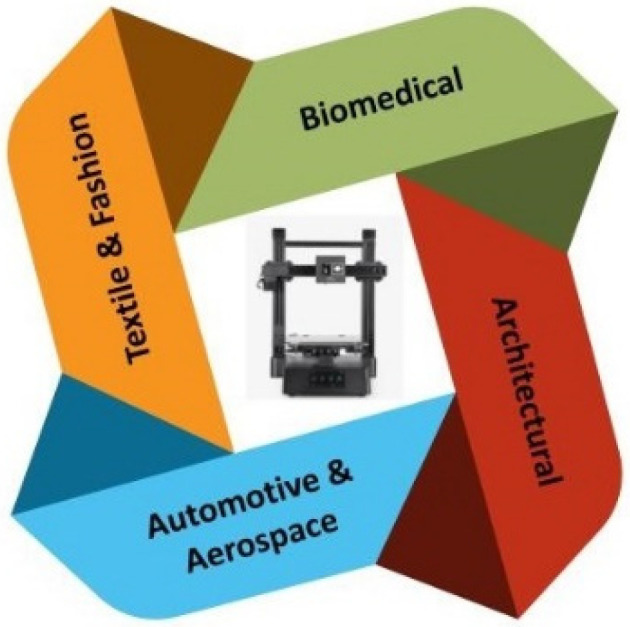
Main areas of FFF application.

Due to its various benefits, there are numerous applications of FFF printing in many fields, including automotive and aerospace, biomedical, architectural, textile and fashion, and other industries (Figure 7). 

Since the 20th century, the automotive sector has always played an important role in the economies of developed countries. The car has undergone a continuous process of evolution in order to adapt to different technological, social and economic contexts; know-how, technologies, functions and aesthetics have changed over time, leading to the conception of an intelligent vehicle with better performances and greater energy efficiency. In recent years, the use of AM technologies in the automotive sector has increased, especially the use of the FFF technique, which is preferred to other machines, due to its versatility, the possibility of producing even final components and the simplicity of the process [48,50]. For example, FFF is used for the production of components with complex geometries, and for rapid prototyping (Research & Development), reducing production times [48]. Some companies use 3D printing to manufacture and decode spare parts at low volume or, in addition, to produce racing cars, parts of Formula 1 car, tools and molds [50]. The molds used in this field are usually fiberglass molds with a thin layer of gelcoat or metal molds. This type of molds presents several disadvantages: they are very expensive, need a long time for their manufacture, and involve the use of release agents during the demolding operations [80,91]. The combination of FFF printing and of chemical sanding was proposed as a solution to produce low-cost molds with an excellent surface finish [50,80,91]. For example, Kuo et al. (2017) indicate a polishing mechanism for ABS parts, based on acetone vapors, obtaining a 98% reduction in roughness [122]; Romero et al. (2021) suggest the use of limonene to chemical polish of high impact polystyrene (HIPS), obtaining an excellent finish of the pieces and overcoming the problems of demolding of FFF molds [80]. Overcoming some limitations, FFF technology produces lighter car components, which results in a reduction in vehicle weight, better performance and lower energy consumption, supporting sustainability. 

Now, FFF printers are also a valuable aid to aerospace manufacturers, researchers and designers. The Fused Filament Fabrication technique has been used for different aerospace applications [50,123]. For example, computational fluid dynamics have been improved and the models have been experimentally validated. In recent years, many aircraft components (such as micro-frames or interior furniture components), as well as repair parts, have been produced using additive manufacturing technologies, but strict manufacturing standards must be adhered to in order to certificate the products [124]. Many steps forward have been taken. For example, some companies are supplying certified printers, filaments and materials, such as the company Stratasys (USA) which has produced a filament based on ULTEM 9085 resin, compliant with the FST standard) and increasingly aircraft manufacturers are using FFF technology within their production process: this means a lighter aircraft and more efficient use of fuel. Moreover, this technology is used for the construction of structural prototypes for Unmanned aerial vehicles (UAVs). UAVs have evolved considerably since their first appearance in the First World War. Interest in UAVs is not only military, but they are also used in civil applications. It was also possible to produce multi-material components and metamaterials, which have unique properties (for example conductive properties). The connube of use of this materials and of the FFF printing reduces the wiring on the aircraft and allows the integration of electronic components such as gyroscopes, accelerometer, barometer, and GPS within the frame, improving the aerodynamics of the UAVs, overall [123]. Thus, complex structures and embedded electronics of UAVs were built successfully through FFF technology [123].

Other applications of FFF relate to the biomedical industry [50,125]. Biomedicine is the field in which the potential of 3D printers has so far developed with extreme effectiveness. For example, FFF is used to produce surgical instruments, which are commonly made of steel. With the technical FFF it is possible to use materials such as ABS, PLA, PA, also with reinforced matrix, for printing sterilized medical devices [15,16,17,125,126]. Some researchers have developed a new method for 3D printing of living skin using built-blood vessels [125]. Furthermore, the customization of geometries and the use of biocompatible materials has led to the production of prostheses and implants [50]. Different type and geometry of scaffolds are actually produced, used as supports for the human tissue growth. FFF technology can provide a higher reproducible control of the size and distribution of pores required for tissue engineering applications and can provide a considerable variety of biodegradability and functionalized materials [50]. A recent development in 3D printing technology in tissue engineering concerns the development of new bio-inks (using collagen, gelatin or hyaluronic acid); this has made it possible to bioprint complex tissue structures [125]. 3D printing is also used for the fabrication and reconstruction of anatomical parts such as bones, vertebrae and spinal implants, prostheses, skin, organs, etc. It enables replacement body organs to be printed to treat specific injuries or defects caused by accidents or diseases. It allows replacement body organs to be printed to treat specific injuries or defects in a patient due to accident or disease. In recent years, the use of 3D printing in the cardiovascular biomedical sector, especially in congenital heart disease, has increased dramatically. It is thus possible to reconstruct the heart, valves, and other anatomical parts of the patient by perfectly studying the complex cardiovascular situation [125]. 

Innovative applications in biomedicine concerns the use of FFF to produce transdermal patches. For example, these are used to treat cancer and tuberculosis, by alleviating problems associated with active drug ingredients [125]. 3D printed patches are commonly used for the delivery of molecules such as antibiotics, growth factors, biometals, etc. [50]. The most commonly used materials for 3D printing are purified metals and ceramics, special polished polymers, and well-checked hydrogels [125]. Moreover, due to the SARS-CoV-2 emergency, many companies but especially makers are turning their business into 3D printed production of face masks, gowns, personal protective equipment, face shields, swabs, splitters, valves, ventilator devices etc. [127].

FFF printing is also gaining popularity in the textile and fashion industry. Clothes, shoes, jewelry and other accessories are now being produced using 3D printing technologies [50]. Scientific studies on the FFF technique show that it offers excellent possibilities for use, thanks to the ease of design and the reduction in production times. Manufacturers have produced knitted fabrics, textiles and garment parts with improved physical and mechanical properties compared to traditional materials, using PLA and softened PLA together with other materials (e.g., Ninjaflex, BendLay, TPE) [50,128]. Research is now also looking at reinforcing materials with PLA, ABS or PC and at improving process parameters, with the aim of improving durability and polymer-fabric adhesions [50,128,129]. The possibility of using FFF filaments made with green and recycled materials could be a valid use in the fashion sector, increasingly attentive to the issues of ecology and sustainability.

The adoption of 3D printing technology in the architectural sector appears more complex than in other sectors, due to the high costs of investment in innovation and development, the regulatory framework, and the reduced number of printing machinery manufacturers [50,130]. Despite this, the main experiments in architecture have focused over the last 20 years on the transition to digital of the building manufacturing process and building components, with particular reference to time and cost savings. In this context, the first layer manufacturing systems were developed in the early 1990s by the Japanese Shimizu Corporation to explore alternative ways of building skyscrapers. Khoshnevis’s research at the University of Southern California followed soon after, leading to the patenting of the Contour Crafting system, a reference model for experimental Construction 3D Printing systems. The most recent experiments concern 3D printing processes mainly in concrete, as in the case of the achievements of the Chinese company WinSun [50], or the Italian WASP (World’s Advanced Saving Project) focused on the development of open-source 3D printing, capable of printing ceramics and porcelain [130]. In contrast, FDM technology necessarily requires the use of high-performance polymeric materials (e.g., PEI, polyaryletherketone PAEK and polyphenylsulfone PPSU). Such materials are increasingly sought after in the construction industry, as they are cheaper and lighter than the stainless steel or aluminum used in the industry. However, the latest research in the construction field is moving in the direction of using FFF printing and the production of composite materials reinforced with stone powders, even better if they are waste and recycled [118,119]. In Table 5, a list of the main FFF application areas with the related specific uses is reported.

D pri3nting is also revolutionizing the field of Cultural Heritage (CH). AM technologies allow to create replicas of different kind of CH, such as archaeological finds, sculptures, architectural elements, paintings and works of art, in general [132,133,134]. The purposes can be multiple (Table 6).

For example, a tangible 3D printed copy can replace an art object, which for various reasons, has to be removed from its original environment. The replacement can be limited in time, such as the loan of a work for a temporary exhibition or for restoration, or again permanent, such as the removal of a statue from its original location for preservation. In this way, the visitor can appreciate the work in the original context in which it was created, while at the same time, the original object is preserved and protected [135]. There are many museums that are already using 3D printing for these purposes. For example, to mark the refurbishment of the Cast Courts in 2018, the V&A commissioned Rapidform at the Royal College of Art produced 3D scans of 23 objects in the collections [135]. In addition, a great deal of scientific research on the advantages/disadvantages of the FFF printing process and materials used in the CH field has been published in recent years. To name the most recent ones, Chatterjee and Dhande (2021) in their paper used digitization and 3D printing to reproduce copies of Indian artifacts [133]. In particular, the Buddhist deities Hariti and Gajlaxmi are studied, and a rapid prototype of the Buddha statue was developed using the FFF technique. Nagy (2021), in his work, deals with the topic of replication as a means of preservation, taking into consideration “The Wages of Sin”, made in 1987 by Mike Kelley and located at The Whitney Museum of American Art (New York) [136]. The author describes the entire process of making the copy in its complexity, analyzing all the critical issues. The candles were 3D printed with ABS filament through the use of FDM printers, in addition to the use of the SLS technique [136]. Bonora et al. (2021) explored the use of the structure from motion (SfM) technique together with FFF printing for the creation of copies of marble statues, to replace the originals for conservation purposes [134]. Their work is presented as an accurate excursus on parameters, advantages and criticalities, through a practical example of creation of a copy of the marble statues of the baptistery of Giovanni in Corte in Pistoia (Italy) [134]. 

Replicas are usually made using molds, which are then filled with resin and gypsum to obtain a copy of the art or archeological object. Contact between the surface of the mold and the artwork is therefore required, but this can affect the conservation process. There are now many software, tools and new technologies that support 3D printing in the reconstruction of works of art, without touching and damaging the artistic surface. For example, digital 3D models can be faithfully reproduced by using laser scanning or photogrammetry [134,152]. 

In order to achieve a high quality reproduction, the SLS technique with marble powder was often used in the beginning for this applications [50]. If plastic materials are used, the object will have an artificial appearance, but in order to have a replica with similar characteristics to the original, technologies and instruments, that allow the use of hybrid materials, must be used. The result of an FFF print is not always comparable in quality to that obtained with other techniques involving lasers, such as SLS, but quality/price ratio is certainly an attractive aspect. For this reason, the FFF technique was mainly not used at first, as it was only based on the use of polymer filaments (i.e., PLA, ABS …). The increasing development of innovative materials and composites has subsequently led to greater use of this technique. However, many steps forward still need to be taken in the field of CH, and a real use of recycled materials is still missing. Today, there are several applications of the FFF technique in the field of conservation and restoration, due to the ease of the process, low costs and continuous developments in the world of research of innovative materials [153,154].

The creation of museum replicas and web advertising have become fairly standard activities of many museums. These activities aim to attract new tourists and also prevent the exclusion of people, who for various reasons, cannot visit the museum in the traditional form, e.g., for economic reasons or security reasons related to the COVID-19 pandemic [154]. For example, the Smithsonian Museum (Washington) has started to share some scans on its website; the British Museum (London) uses a platform (called Sketchfab) that allows anyone to share or sell their 3D models. In Italy, the first Italian online three-dimensional museum called “3D Virtual Museum” was born, which brings together some collections of various Italian museums. The works of art published on the site are described by an information sheet, and some can be downloaded in STL format and printed in 3D [154]. 

Additive Manufacturing techniques are not only suitable for the reproduction of copies of small/medium size works of art (such as statues, archaeological finds, paintings...) but also for the realization of large scale works, such as archaeological sites and architectural monuments. Fotia et al. (2021) reproduced a 1:50 scale 3D model of the Saracen Tower Remains, located at the entrance of the ancient city of Cardeto (Reggio Calabria, Italy) from the 10th–11th centuries, using the “Bq Hephestos 2” printer and the advantages of the FDM technique [141]. Polylactic acid was selected by the authors as the material for printing, due to its relatively low melting temperature, low shrinkage index, discrete mechanical properties and non-toxicity, compared to other materials, such as ABS. Clini et al. (2017) described the process of 3D reproduction of the Arch of Trajan, one of the most precious monuments built in Ancona (Italy) in 100–115 AD by Apollodorus of Damascus, in honor of Emperor Trajan [155]. The main objectives were to improve the fruition (since the access to the area where the monument is located has been limited for security reasons in recent years) and to give the possibility to all visitors to observe every single detail. Through an elaborated study that has foreseen the TLS survey and the creation of the CAD model (.dwg file), the printing of the 3D model in scale 1:50 (dimensions 22.4 × 9.6 × 27.6 cm) has been finally obtained. ABS was used as printing material along with FDM technique (Fortus 250mc printer). Despite the small size of the printed object, AM allowed the printing of the most important details, reproducing quite finely even the decoration of the arch [155].

It is necessary to consider that 3D printing of architectural monuments reduces the entire 3D model by up to 200 times. Many important architectural and ornamental details may in fact be lost with this scaling of the 3D model, in some cases. Montusiewicz et al. (2021) in their manuscript expose critical issues and advantages of 3D printing of architectural structures [140]. They describe the various steps of the multi-level procedure, giving some examples of 3D copies of monuments, located in Lublin (Poland). The real model is decomposed into submodels by this procedure and different scale values for individual elements are used, preserving also excellent decorative details of the original monuments [140].

By combining 3D scanning and printing technologies, it is not only possible to scale large artifacts, but also to magnify microscopic objects [142]. This can be useful for morphometric studies in various areas of science, from anatomy, zoology, anthropology, paleobotany, and paleontology [139,143]. 3D reproduction of fossils, for example, has become a valuable tool for taxonomic placement of the specimen and is a very important development to enhance and make visible to the general public the research carried out by various institutes [142]. 

3D printing technologies can also contribute to the restoration of works of art. Many sculptures and monuments are preserved with essential missing parts, which can be replaced by artificial copies to give to the visitors a full explanation of what the original structure looked like. FFF technology can accurately and quickly reproduce the lacuna in many type of artistic objects. Higueras et al. (2021) demonstrated the validity of 3D printing for non-invasive restoration applications, dealing with the virtual reintegration of a missing part of a Roman cornice from Castulo Archaeological Site (Spain) [144]. The missing fragment was constructed by the use of a mold, obtained using photogrammetry, FFF printing, and PLA. In this way, an excellent surface resolution of the floral decoration of the cornice was obtained, with a reduction in costs and no manipulation of the original object. In addition, the use of PLA made it possible to have a slight and reversible object, and to apply a polychrome finish later. In fact, today the possibility of using FFF printer filaments of a wide variety of colors and inerts in the field of CH, as well as of coloring commercial PLA, makes it possible to meet the different needs of artwork conservation. Numerous steps forward have been made since the first experiments of reintegration of missing parts, when for example in 2014, the Heritage Lab working group reconstructed the heads of the two little angels of the side chapels of the Church “Castello di San Martino dall’Argine” (Mantova, Italy). Through the use of open source image-based techniques (Python Photogrammetry Toolbox and Meshlab) and an FFF printer (Coobot 3D-PR model with double extruder) they were able to perform the reintegration with relatively low costs, but the yellow color of the heads of the angels conferred by the polymer used in the printing, did not perfectly integrate with the rest of the artwork (https://www.digitalmeetsculture.net/article/3d-printing-applied-to-cultural-heritage/?upm_export=pdf, accessed on 30 November 2021). The restored parts must certainly be distinguishable from the original, but without disturbing the entire view of the work of art. On the other hand, it must also be considered that PLA is a highly hygroscopic material, so its poor durability suitable for the field of restoration (reversibility is another basic principle of reintegration) may not always be ideal for all CH applications.

Digital fabrication technologies can be widely used to create customized packaging or support structures for the storage, transport or display of fragile artefacts. The risks involved in handling objects of great cultural value are greatly reduced. The most suitable materials can be chosen to preserve the object, thus reducing production costs. Moreover, 3D printing can also be used to create appropriate support structures to give visitors a good fruition of the artistic find in museums [134]. To cite an example, Fatuzzo et al. (2017) produced the ABS packaging for the bronze statue of Heracles (Museo Civico F.L. Belgiorno, Modica, Italy), using a Stratasys 3D printer (Dimension 1200es model), through the FDM technique [145].

The creation of replicas can also be useful for building alternative museum itineraries and in education. In fact, the possibility of interacting with objects through contact is not always possible because museums often display unique and valuable pieces. This modern museum practice limits visitors’ understanding of the collections, granting only a unisensory visual experience. In contrast, recent research has shown that the ability to touch objects in museums and heritage sites allows us to understand the world around us in a new way, and that these tactile experiences enchant and excite visitors [147].

These limitations are easily overcome thanks to 3D scanning and plastic printing, and so numerous projects, companies and new museum itineraries was born in recent years. For example, interactive education is used at the American Museum of Natural History of New York, where there is an itinerary called “Capturing Dinosaurs: Reconstructing Extinct Species Through Digital Fabrication” where thanks to 3D printed scale models of different dinosaurs, school students have been able to approach the profession of paleontologist. Another example is represented by the exhibition of a showcase dedicated to the evolution of man inside the “Archeologico Museo di Massa Marittima” (Tuscany, Italy), where a series of seven replicas of various fossil finds (from Australopithecus to Homo) has been entirely realized with the use of the FFF printer and the application of color for the surface finishing on the thermoplastic material. The series of Mesolithic headdresses from the Star Carr site in Yorkshire is another example of application in this field, which were 3D printed for an exhibition at the Museum of Archaeology and Anthropology in Cambridge (MAA). This overcame the problems associated with the fragility of the original headdresses, allowing visitors to understand the artefacts. The direct experience and the manipulation of the finds, especially in young people, has a very important role in learning.

FFF technology has now also been acquired within museums and cultural foundations, giving rise to innovative laboratories equipped with scanners and 3D printers, which are dedicated to enhancing cultural heritage and promoting educational learning and social inclusion. This has undoubtedly been aided by the fact that FFF technology is user-friendly, so knowledge is easily transferable, and that FFF printers require little space. An example is represented by the “MArTA Lab”, a laboratory located at the “Museo Archeologico Nazionale di Taranto-MArTA” (Puglia, Italy) that deals specifically with the conservation of archaeological heritage and carries out activities, such as teaching for schools and courses for all on open source 3D printing, museum merchandising, as well as the reproduction of true copies for the creation of new exhibition spaces. The “Fondazione Museo Civico” of Rovereto (Italy) is also equipped with the “Laboratorio di modellazione e stampa 3D”, among the several labs present inside (e.g., geophysics, dendrochronology, archaeology...), which uses the uPrint3D printer (FFF methodology), to promote the fruition of the finds and the educational dissemination. The “Museo delle Scienze” (MUSE) in Trento (Italy) hosts the “Fablab”, a workshop containing a complete set of tools for digital fabrication (3D printers, numerical control milling machines, vinyl cutting machines, laser cutters, etc.), as well as a workstation for electronic processing, a wall equipped for analog processing and a small library of essays and manuals related to the world of “making”. “The Museum of Science and Industry” in Chicago has the “Wanger Family Fab Lab”, a small workshop where anyone can dream, design and print objects (including souvenirs) using modern software and equipment, including FFF. 3D printing generates attraction in the public and can also be an incentive to fruition, when integrated within existing museum environments.

3D printing has generated a new business through museum merchandising [150,151]. Today, these methodologies represent an additional tool for the dissemination of the image, heritage and related cultural message of the institution, as well as an increase in the financial resources of museums. In this sector, through instrumentation for 3D acquisition and printing, it is possible to create high definition molds to be used for the mass production of small copies of the original [138,142].

Moreover, 3D printed objects can be very useful in helping blind people visualise sculptures or artwork through touch, without direct contact with the original surface. This can be done simply by producing replicas with designs adapted to the perception of the surface details of the work. Some interesting studies have applied these methodologies not only to transform statues and archaeological finds, but also photographs and paintings. For example, the cooperative society of services for CH called “ArcheoLab” (Italy) has reproduced in 3D printing the portrait of the poet Luigi Groto by Tintoretto and the famous Mona Lisa by Leonardo da Vinci, to make the paintings accessible through touch, even to blind and visually impaired visitors. An example of sensory museum accessibility dedicated to the disabled has been created at the Museum of Prehistory “Paolo Graziosi” in Florence. The objective of the work was to make accessible to the blind and deaf two important historical artifacts conserved in the museum: the Venus of Laussel of about 20,000 years ago and the Lion Man of Hohlenstein of about 40,000 years ago. The prehistoric artifacts were 3D printed, accompanied by a panel with a Braille description and an audio device. In this way, a multisensory experience was created, touching the reproductions and listening to their description (paleos.it). In Florence (“Sala della Balena” at the “Museo di Storia Naturale”), in 2015, fossils of marine animals, belonging to the Tuscan marine ecosystem of 3million years ago were reproduced with the FFF technique. In order to create a didactic itinerary that would involve the public with special needs, particularly the visually impaired, the finds were printed with non-toxic plastic (PLA), favoring bright colors that would clearly distinguish the tactile aid from the find. 

Numerous national and international projects have emerged in recent years with the aim of involving people with special needs and disabilities. For example, the project “Cooperating for open access to museums towards a wider inclusion” (COME-IN!) addressed this issue by increasing the capacity of European museums to make them accessible to a wider audience of people with different types of disabilities. Or the project “We Encourage Living Collective Open Museums Experiences” (WELCOME) which involved eight museums in the region of Tuscany (Italy) with activities of social inclusion and intercultural integration, based on the introduction of new multisensory paths with the use of 3D printing, and many others.

## 5. Conclusions

This paper aimed at giving a detailed excursus of the recent advances on innovative polymer-based materials for Fused Filament Fabrication (FFF), including commercial, composite and nanocomposites, natural and recycled filaments. All kinds of possible industrial applications of FFF technique, even when innovative filaments were used, are also analyzed. Finally, the field of Cultural Heritage applications was deeply studied, evidencing advantages, limits, and potential future perspectives. The analysis showed that the quality of the FFF printed models is not always comparable to that obtained with other AM techniques, such as SLS. However, FFF is certainly the most economic technique. Another issue related to the application of FFF for Cultural Heritage field is the use of commercial standard polymer filaments (i.e., PLA, ABS...), that seem to be still inappropriate to adequately reproduce artwork. On the other hand, the growth of the availability of innovative composites and nanocomposites filaments for FFF, has determined an increasing use of this technique. However, many issues still need to be overcome to improve the final aspect of the restored parts. In addition, the use of recycled polymer-based filaments is still missing in the field of CH. The most used filament remains PLA or even PLA based composites. However, it is a highly hygroscopic polymer, characterized by a weak durability, if exposed to outdoor conditions, due to the synergic effect of the humidity of air, temperature, and UV exposure. This latter problem makes PLA non completely suitable for all CH applications. In this context, the current research activity of the authors is inserted. We are, in fact, studying the possibility to develop an original method to produce composite filaments, consisting of PLA and industrial wastes (based on wood, ceramic, stone, etc.). The filaments will be used to produce design objects by means of a low cost FFF machine. In order to preserve the printed models from aging, they will be properly treated with super hydrophobic green coatings, patented by some of the authors.

## Figures and Tables

**Table 1:** Properties of some common polymers used in FFF technology.

Polymer	Melting Temperature (°C)	Tensile Strength (MPa)	Young’s Modulus (GPa)	Biodegradability	Filament Diameter (mm)	Extrusion Temperature (°C)	Printing Speed (mm/s)	Ref.
ABS	177–320	11–65	1–2.65	NO	1.75 ± 0.05	215–275	25–40	[45,46,47,48,49,50]
PLA	120–205	30–65	2.3–2.9	YES	1.75 ± 0.05	160–230	25–110	[45,46,48,49,50]
PEEK	343	100	3.56–4.00	NO	1.75 ± 0.05	340–440	5–30	[45,48,50,51]
PETG	230–260	29–56	0.42–0.88	NO	1.75 ± 0.05	220–250	15–45	[45,46,48,49,50,52]
PC	250–343	58.6–72	1.79–3.24	NO	1.75 ± 0.05/1.75 ± 0.07	200–280	25	[49,53]
PA	216–300	35–186	0.45–3.50	NO	1.75 ± 0.05	230–250	40	[48,49,50]
HDPE	120–220	24.5–27.5	0.88–1.10	NO	1.75 ± 0.05/2.8 ± 0.01	200–260	25–250	[46,48,54]
PP	150–160	17–21	0.79–0.88	NO	1.75 ± 0.05	165–250	8–60	[46,48,55,56]

**Table 2:** Properties of some composites used in FFF technology.

	Polymer	Filler	Content of Filler (%)	Tensile Strength (Mpa)	Young’s Modulus (GPa)	Filament Diameter (mm)	Extrusion Temperature (°C)	Printing Speed (mm/s)	Ref.
micro and nanoparticles	PLA	Cu, Al, graphene	1.6–4	15–40	/	/	180–210	/	[64]
	LDPE	glass	30	/	0.22	1.45 ± 0.10	210	3	[65]
	ABS	TiO_2_	5	18.4–32.2	1.35–1.71	1.9	230	40	[66]
	PP	glass	30	8.1–20.6	1.05–1.65	1.9	/	/	[67]
	ABS	BaTiO_3_	10–35	13.7–25.5	2.6–3.3	1.75 ± 0.10	210–230	/	[68]
	Nylon	Fe	30–40	/	/	1.78–1.85	/	/	[69]
	PEG-PVB and silicone gel	Fe_3_O_4_	20–60	/	/	/	/	5–10	[70]
	ABS	graphene	20	30	2.4	1.75	220	20	[71]
	ABS	Cu	10–50	26.5–42	0.9	1.75	/	/	[72]
	PLA	hydroxyapatite	3.4	/	3	1.75	200	50	[16]
	PLA	hydroxyapatite	30	/	/	1.75	150	30	[17]
	PP	carbon black	15.5–32.3	/	/	1.4–1.7	230	/	[73]
fibers	Nylon	glass fiber	/	156–212	3.28–4.91	/	263	/	[74]
	Nylon	carbon fiber	/	198	8.46	/	263	/	[74]
	Nylon	evlar fiber	/	110–161	4.23–4.76	/	263	/	[74]
	PP	glass fiber	30	28–45	1.4–2.2	1.75	185	8	[75]
	PETG	CNT	/	46	1.79	1.75	230	5–10	[76]
	PEEK	CNT	1–5	65–100	/	2.7 ± 0.3	350–390 °C	30	[77]
	PLA	AgNW	1–4	/	/	1.75	210	/	[78]

**Table 3:** Examples of some filaments for FFF based on natural materials.

Material Composition		Filament Diameter (mm)	Extrusion Temperature (°C)	Printing Speed (mm/s)	Tensile Strength (MPa)	Young’s Modulus (GPa)	Ref.
natural material	PLA/low-cost kraft lignin (5%)	1.78 ± 0.04	205	20	40.8–51.2	2.28–2.47	[95]
	PLA/cork/TBC	1.75 ± 0.05	230	30	30.53 ± 1.0	2.49 ± 0.15	[96]
	ABS or PLA/beech wood (0–50%)	1.75–1.45	275 (ABS), 230 (PLA)	30	30–57	3.0–3.94	[47,97]
	PLA/pine wood/rice husk	1.75	210	/	30–40	1.5–2.0	[98]
	PLA/bamboo powder/PEG	1.8	175, 195	30, 50	/	/	[99]
	PLA/hemp and harakeke	3/2.6–3	110	/	24–30	2.7–4.2	[100]
	ABS/rice straw (5–15%)	1.75 ± 0.3	250	/	12–30	1.3–2.5	[101]

**Table 4:** Examples of some filaments for FFF based on recycled materials.

Material Composition		Filament Diameter (mm)	Extrusion Temperature (°C)	Printing Speed (mm/sec)	Tensile Strength (MPa)	Young’s Modulus (GPa)	Ref.
recycle material	recycled PET from bottles	1.75	250	50	33.79–47.08	0.65–1.36	[112]
	recycled Nylon-6/ABS/TiO_2_	1.75	235, 230	50, 40	76.20–86.91	1.64–2.34	[113]
	recycled packaging APWW/EG	1.75	165–190	2.5	13.58	/	[114]
	recycled cellulose/PLA	/	190	60	32.71–38.74	2.00–2.8	[115]
	recycled Agave leaves/PLA	1.7 ± 0.07	190	50	28–51	2.5–3.4	[116]
	wind turbine waste/PLA	1.75	215	40	41.94–57.57	3.17–4.03	[117]
	recycled marble dust/PLA	/	195	/	49.1–53.08	2.69–3.83	[118]
	Lecce stone waste/PLA	1.75	200	50	/	/	[119]

**Table 5:** FFF application areas and specific uses.

Sector	Specific Use	Ref.
Automotive	prototypes, research and development, molds, racing cars, tools, components of cars	[80,91,122,131]
Aerospace	prototypes, research and development, computational fluid dynamics, micro-frames or interior aircraft components, repair parts, unmanned aerial vehicles, electronic element integration components	[50,63,123,124]
Biomedical	prototypes, research and development, microdevices, surgical tools, medical device, personal protective equipment, face masks, prostheses, implants, tissue engineering, scaffolds and bio-ink, reconstruction of anatomical parts, transdermal patches, drug delivery system, valves, ventilator devices	[15,16,17,18,19,20,34,50,86,125,126,127]
Textile and fashion	prototypes, research and development, fabrics, clothes, shoes, jewelry, accessories, ornaments	[50,128,129,131]
Architectural	prototypes, research and development, buildings, building components	[50,118,119,130]

**Table 6:** Applications of FFF in the field of CH.

Aim	Specific Use	Ref.
Research and development	production of new composite filaments and prototypes	[50,119,134,135]
Permanent or temporary replacement of artwork	replicas of archaeological finds, sculptures, architectural elements, paintings and works of art and molds	[133,134,136,137]
Fruition by web	online museum collections of 3D replicas, sharing of cad and stl models	[138,139]
Historical or morphometric studies	reconstruction of archaeological sites, monuments, archaeological finds, fossil	[140,141,142,143]
Restoration	reintegration of missing parts	[142,144]
Storage, transport or display of fragile artefacts	customized packaging or support structures	[134,145,146]
Fruition, conservation and education	new museum itineraries with touchable replicas of works of art	[139,142,147,148,149]
Valorization and promotion of educational learning	innovative laboratories equipped with scanners and 3D printers located in the museum	[139]
Promotion and new business	museum merchandising, souvenir	[139,142,150]
Fruition and social inclusion	new multisensory museum itineraries (3D replicas of artworks, panel with a Braille, audio device)	[139,149,151]

## Data Availability

Not applicable.

## Nomenclature

ABS	Acrylonitrile Butadiene Styrene
AM	Additive Manufacturing
AMF	Additive Manufacturing Format
APC	AcetoxyPropyl Cellulose
ASTM	American Society for Testing Materials
ATR-FTIR	Attenuated Total Reflectance[––]Fourier Transform Infrared Spectroscopy
BFs	Basalt Fibers
BJ	Binder Jetting
CA	Cellulose Acetate
CAD	Computer-Aided Design
CH	Cultural Heritage
CN	Cellulose Nanocells
CNFs	Cellulose NanoFibers
CSW	Cocoa Shell Waste
μCT	Micro-Computed Tomography
2D	Two-Dimensional
3D	Three-Dimensional
4D	Four-Dimensional
DDM	Direct Digital Manufacturing
DED	Directed Energy Deposition
DLP	Digital Light Processing
DOD	Drop On Demand
DSC	Differential Scanning Calorimetry
EBM	Electron Beam Melting
EG	Exfoliated Graphite
EPDM-g-MAH	Ethylene Propylene Diene Monomer grafted with Maleic AHydride
FDM	Fused Deposition Modelling
FFF	Fused Filament Fabrication
FRP	Fiber Reinforced Polymers
HDPE	High Density PolyEthylene
HIPS	High Impact PolyStyrene
HPPM	High-Performance Polymeric Materials
LCD	Liquid Crystal Display
LDPE	Low Density PolyEthylene
LENS	Laser Engineered Net Shaping
LLDPE	Linear Low Density PolyEthylene
LM	Layer Manufacturing
LOM	Laminate Object Manufacturing
LS	Lecce Stone
MD	Marble Dust
MDI	DiphenylMethyl propane diisocyanate
ME	Material Extrusion
MJ	Material Jetting
MJM	Multi Jet Modelling
MOE	Modulus Of Elasticity
MPa	Mega Pascals
OPEFB	Oil Palm Empty Fruit Bunch
OPF	Oil Palm Fiber
PA	PolyAmide
PAEK	PolyArylEtherKetone
PBF	Powder Bed Fusion
PBS	PolyButylene Succinate
PC	PolyCarbonate
PCL	PolyCaproLactone
PMC	Polymer Matrix Composite
PE	PolyEthylene
PEG	PolyEthylene Glycol
PEI	PolyEtherImide
PEEK	PolyEtherEtherketone
PET	PolyEthylene Terephthalate
PETG	PolyEthylene Terephthalate Glycol
PHA	PolyHydroxyAlkanoates
PLA	Polylactic Acid
PMC	Polymer Matrix Composites
PMMA	PolyMethyl MethylAcrylate
PP	PolyPropylene
PPSU	PolyPhenylSUlfone
PS	PolyStyrene
PVA	PolyVinyl Alcohol
PVC	Polyvinyl Chloride
R&D	Research and Development
RH	Relative Humidity
RM	Rapid Manufacturing
RP	Rapid Prototyping
RT	Rapid Tooling
SEM	Scanning Electron Microscope
SL	Sheet Lamination
SLA	Stereo Lithography
SLM	Selective Laser Melting
SLS	Selective Laser Sintering
STL	Standard Triangulation Language
TBC	TriButyl Citrate
TGA	ThermoGravimetric Analysis
TPE	ThermoPlastic Elastomer
TPU	Thermoplastic PolyUrethane
TRIP	Techniques Recovery Innovative Printable
UAVs	Unmanned Aerial Vehicles
UF	Urea Formaldehyde
UV	UltraViolet
VP	Vat Photo Polymerization
WF	Wood Flour
XRD	X-ray Diffraction

## References

[1] Wang X., Jiang M., Zhou Z., Gou J., Hui D. (2017). 3D printing of polymer matrix composites: A review and prospective. Compos. Part B Eng..

[2] Daminabo S.C., Goel S., Grammatikos S.A., Nezhad H.Y., Thakur V.K. (2020). Fused deposition modeling-based additive manufacturing (3D printing): Techniques for polymer material systems. Mater. Today Chem..

[3] Wu H., Fahy W.P., Kim S., Kim H., Zhao N., Pilato L., Kafi A., Bateman S., Koo J.H. (2020). Recent developments in polymers/polymer nanocomposites for additive manufacturing. Prog. Mater. Sci..

[4] Ivanova O., Williams C., Campbell T. (2013). Additive manufacturing (AM) and nanotechnology: Promises and challenges. Rapid Prototyp. J..

[5] González-Henríquez C.M., Sarabia-Vallejos M.A., Rodriguez-Hernandez J. (2019). Polymers for additive manufacturing and 4D-printing: Materials, methodologies, and biomedical applications. Prog. Polym. Sci..

[6] Tofail S.A.M., Koumoulos E.P., Bandyopadhyay A., Bose S., O’Donoghue L., Charitidis C. (2018). Additive manufacturing: Scientific and technological challenges, market uptake and opportunities. Mater. Today.

[7] Blok L.G., Longana M.L., Yu H., Woods B.K.S. (2018). An investigation into 3D printing of fibre reinforced thermoplastic composites. Addit. Manuf..

[8] Melnikova R., Ehrmann A., Finsterbusch K. (2014). 3D printing of textile-based structures by Fused Deposition Modelling (FDM) with different polymer materials. IOP Conf. Ser. Mater. Sci. Eng..

[9] Christ J.F., Aliheidari N., Ameli A., Pötschke P. (2017). 3D printed highly elastic strain sensors of multiwalled carbon nanotube/thermoplastic polyurethane nanocomposites. Mater. Des..

[10] Rigotti D., Dorigato A., Pegoretti A. (2018). 3D Printable Thermoplastic Polyurethane Blends with Thermal Energy Storage/Release Capabilities.

[11] Nelson M.D., Ramkumar N., Gale B.K. (2019). Flexible, transparent, sub-100 μm microfluidic channels with fused deposition modeling 3D-printed thermoplastic polyurethane. J. Micromech. Microeng..

[12] Korpela J., Kokkari A., Korhonen H., Malin M., Narhi T., Seppalea J. (2013). Biodegradable and bioactive porous scaffold structures prepared using fused deposition modeling. J. Biomed. Mater. Res. Part B Appl. Biomater..

[13] Walker J.L., Santoro M. (2017). Processing and Production of Bioresorbable Polymer Scaffolds for Tissue Engineering.

[14] Chen X., Gao C., Jiang J., Wu Y., Zhu P., Chen G. (2019). 3D printed porous PLA/nHA composite scaffolds with enhanced osteogenesis and osteoconductivityin vivo for bone regeneration. Biomed. Mater..

[15] Esposito Corcione C., Gervaso F., Scalera F., Padmanabhan S.K., Madaghiele M., Montagna F., Sannino A., Licciulli A., Maffezzoli A. (2019). Highly loaded hydroxyapatite microsphere/PLA porous scaffolds obtained by fused deposition modelling. Ceram. Int..

[16] Corcione C.E., Gervaso F., Scalera F., Montagna F., Maiullaro T., Sannino A., Maffezzoli A. (2017). 3D printing of hydroxyapatite polymer-based composites for bone tissue engineering. J. Polym. Eng..

[17] Corcione C.E., Scalera F., Gervaso F., Montagna F., Sannino A., Maffezzoli A. (2018). One-step solvent-free process for the fabrication of high loaded PLA/HA composite filament for 3D printing. J. Therm. Anal. Calorim..

[18] Makvandi P., Corcione C.E., Paladini F., Gallo A.L., Montagna F., Jamaledin R., Pollini M., Maffezzoli A. (2018). Antimicrobial modified hydroxyapatite composite dental bite by stereolithography. Polym. Adv. Technol..

[19] Dias D., Vale A.C., Cunha E.P.F., Paiva M.C., Reis R.L., Vaquette C., Alves N.M. (2021). 3D-printed cryomilled poly(ε-caprolactone)/graphene composite scaffolds for bone tissue regeneration. J. Biomed. Mater. Res. Part B Appl. Biomater..

[20] Silva M., Pinho I.S., Covas J.A., Alves N.M., Paiva M.C. (2021). 3D printing of graphene-based polymeric nanocomposites for biomedical applications. Funct. Compos. Mater..

[21] Masood S.H., Song W.Q. (2004). Development of new metal/polymer materials for rapid tooling using Fused deposition modelling. Mater. Des..

[22] Brenken B., Barocio E., Favaloro A., Kunc V., Pipes R.B. (2018). Fused filament fabrication of fiber-reinforced polymers: A review. Addit. Manuf..

[23] Krajangsawasdi N., Blok L.G., Hamerton I., Longana M.L., Woods B.K.S., Ivanov D.S. (2021). Fused deposition modelling of fibre reinforced polymer composites: A parametric review. J. Compos. Sci..

[24] Gao X., Yu N., Li J. (2020). Influence of Printing Parameters and Filament Quality on Structure and Properties of Polymer Composite Components Used in the Fields of Automotive.

[25] Turner B.N., Gold S.A. (2015). A review of melt extrusion additive manufacturing processes: II. Materials, dimensional accuracy, and surface roughness. Rapid Prototyp. J..

[26] Xia H., Lu J., Tryggvason G. (2019). A numerical study of the effect of viscoelastic stresses in fused filament fabrication. Comput. Methods Appl. Mech. Eng..

[27] Wu W.P.H., Fahy S., Kim H., Kim N., Gibson D.I., Rosen B.S., Gibson I., Rosen D.B.S. (2015). Extrusion-based systems. Additive Manufacturing Technologies: 3D Printing, Rapid Prototyping, and Direct Digital Manufacturing.

[28] Fallon J.J., McKnight S.H., Bortner M.J. (2019). Highly loaded fiber filled polymers for material extrusion: A review of current understanding. Addit. Manuf..

[29] Ghisellini P., Cialani C., Ulgiati S. (2016). A review on circular economy: The expected transition to a balanced interplay of environmental and economic systems. J. Clean. Prod..

[30] (2012). Ellen MacArthur Foundation towards the Circular Economy: Economic and Business Rationale for an Accelerated Transition. https://ellenmacarthurfoundation.org/towards-the-circular-economy-vol-1-an-economic-and-business-rationale-for-an.

[31] Kirchherr J., Reike D., Hekkert M. (2017). Conceptualizing the circular economy: An analysis of 114 definitions. Resour. Conserv. Recycl..

[32] Vyavahare S., Teraiya S., Panghal D., Kumar S. (2020). Fused deposition modelling: A review. Rapid Prototyp. J..

[33] Licciulli A., Corcione C.E., Greco A., Amicarelli V., Maffezzoli A. (2004). Laser stereolithography of ZrO_2_ toughened Al_2_O_3_. J. Eur. Ceram. Soc..

[34] Corcione C.E., Greco A., Maffezzoli A. (2004). Photopolymerization kinetics of an epoxy-based resin for stereolithography. J. Appl. Polym. Sci..

[35] Ngo D. Formlabs Form 2 3D Printer Review: An Excellent 3D Printer for a Hefty Price 2016. https://www.cnet.com/reviews/formlabs-form-2-3d-printer-review/.

[36] The Ultimate Guide to Stereolithography (SLA) 3D Printing 2017. https://formlabs.com/blog/ultimate-guide-to-stereolithography-sla-3d-printing/.

[37] On the Difference between DLP and LCD Based SLA Printers 2019. https://www.matter-replicator.com/2019/03/02/on-the-difference-between-dlp-and-lcd-based-sla-printers/.

[38] Gibson I., Rosen D., Stucker B. (2010). Additive Manufacturing Technologies–Rapid Prototyping to Direct Digital Manufacturing.

[39] Thompson M.K., Moroni G., Vaneker T., Fadel G., Campbell R.I., Gibson I., Bernard A., Schulz J., Graf P., Ahuja B. (2016). Design for Additive Manufacturing: Trends, opportunities, considerations, and constraints. CIRP Ann. Manuf. Technol..

[40] Levy G.N., Schindel R., Kruth J.P. (2003). Rapid manufacturing and rapid tooling with layer manufacturing (LM) technologies, state of the art and future perspectives. CIRP Ann. Manuf. Technol..

[41] Medellin-Castillo H.I., Zaragoza-Siqueiros J. (2019). Design and manufacturing strategies for fused deposition modelling in additive manufacturing: A review. Chin. J. Mech. Eng..

[42] Li H., Wang T., Sun J., Yu Z. (2017). Rapid Prototyping Journal the effect of process parameters in fused deposition modelling on bonding degree and mechanical properties (2018) “The effect of process parameters in fused deposition modelling on bonding degree and mechanical properties”. Rapid Prototyp. J. Rapid Prototyp. J. Iss Rapid Prototyp. J..

[43] Mohan N., Senthil P., Vinodh S., Jayanth N. (2017). A review on composite materials and process parameters optimisation for the fused deposition modelling process. Virtual Phys. Prototyp..

[44] Gao X., Qi S., Kuang X., Su Y., Li J., Wang D. (2021). Fused filament fabrication of polymer materials: A review of interlayer bond. Addit. Manuf..

[45] Algarni M., Ghazali S. (2021). Comparative study of the sensitivity of pla, abs, peek, and petg’s mechanical properties to fdm printing process parameters. Crystals.

[46] Rajendran Royan N.R., Leong J.S., Chan W.N., Tan J.R., Shamsuddin Z.S.B. (2021). Current state and challenges of natural fibre-reinforced polymer composites as feeder in fdm-based 3d printing. Polymers.

[47] Kariz M., Sernek M., Obućina M., Kuzman M.K. (2018). Effect of wood content in FDM filament on properties of 3D printed parts. Mater. Today Commun..

[48] Pervaiz S., Qureshi T.A., Kashwani G., Kannan S. (2021). 3D printing of fiber-reinforced plastic composites using fused deposition modeling: A status review. Materials.

[49] Rett J.P., Traore Y.L., Ho E.A. (2021). Sustainable materials for fused deposition modeling 3D printing applications. Adv. Eng. Mater..

[50] Cano-Vicent A., Tambuwala M.M., Hassan S.S., Barh D., Aljabali A.A.A., Birkett M., Arjunan A., Serrano-Aroca Á. (2021). Fused deposition modelling: Current status, methodology, applications and future prospects. Addit. Manuf..

[51] Zanjanijam A.R., Major I., Lyons J.G., Lafont U., Devine D.M. (2020). Fused filament fabrication of peek: A review of process-structure-property relationships. Polymers.

[52] Guessasma S., Belhabib S., Nouri H. (2019). Printability and tensile performance of 3D printed polyethylene terephthalate glycol using fused deposition modelling. Polymers.

[53] Vidakis N., Petousis M., Korlos A., Velidakis E., Mountakis N., Charou C., Myftari A. (2021). Strain rate sensitivity of polycarbonate and thermoplastic polyurethane for various 3d printing temperatures and layer heights. Polymers.

[54] Schirmeister C.G., Hees T., Licht E.H., Mülhaupt R. (2019). 3D printing of high density polyethylene by fused filament fabrication. Addit. Manuf..

[55] Dong M., Zhang S., Gao D., Chou B. (2019). The study on polypropylene applied in fused deposition modeling. AIP Conf. Proc..

[56] Milosevic M., Stoof D., Pickering K.L. (2017). Characterizing the mechanical properties of fused deposition modelling natural fiber recycled polypropylene composites. J. Compos. Sci..

[57] Vidakis N., Petousis M., Velidakis E., Liebscher M., Mechtcherine V., Tzounis L. (2020). On the strain rate sensitivity of fused filament fabrication (Fff) processed pla, abs, petg, pa6, and pp thermoplastic polymers. Polymers.

[58] Mikula K., Skrzypczak D., Izydorczyk G., Warchoł J., Moustakas K., Chojnacka K., Witek-Krowiak A. (2021). 3D printing filament as a second life of waste plastics—A review. Environ. Sci. Pollut. Res..

[59] Cao Q., Xie H. (2017). Characterization for elastic constants of fused deposition modelling-fabricated materials based on the virtual fields method and digital image correlation. Acta Mech. Sin. Xuebao.

[60] Kristiawan R.B., Imaduddin F., Ariawan D., Arifin Z. (2021). A review on the fused deposition modeling (FDM) 3D printing: Filament processing, materials, and printing parameters. Open Eng..

[61] Stoof D., Pickering K. (2018). Sustainable composite fused deposition modelling filament using recycled pre-consumer polypropylene. Compos. Part B Eng..

[62] Shanmugam V., Pavan M.V., Babu K., Karnan B. (2021). Fused deposition modeling based polymeric materials and their performance: A review. Polym. Compos..

[63] Lyons B., Ochsendorf J., Leuthardt E.C. (2000). Frontiers of engineering. Front. Eng..

[64] Ghiban B., Pascu N.E., Antoniac I.V., Jiga G., Milea C., Petre G., Gheorghe C., Munteanu C., Istrate B. (2021). Surface characterization of fracture in polylactic acid vs. PLA + Particle (Cu, Al, Graphene) insertions by 3D fused deposition modeling technology. Coatings.

[65] Olesik P., Godzierz M., Kozioł M. (2019). Preliminary characterization of novel LDPE-based wear-resistant composite suitable for FDM 3D printing. Materials.

[66] Torrado Perez A.R., Roberson D.A., Wicker R.B. (2014). Fracture surface analysis of 3D-printed tensile specimens of novel ABS-based materials. J. Fail. Anal. Prev..

[67] Spoerk M., Savandaiah C., Arbeiter F., Sapkota J., Holzer C. (2019). Optimization of mechanical properties of glass-spheres-filled polypropylene composites for extrusion-based additive manufacturing. Polym. Compos..

[68] Khatri B., Lappe K., Habedank M., Mueller T., Megnin C., Hanemann T. (2018). Fused deposition modeling of ABS-barium titanate composites: A simple route towards tailored dielectric devices. Polymers.

[69] Masood S.H., Song W.Q. (2005). Thermal characteristics of a new metal/polymer material for FDM rapid prototyping process. Assem. Autom..

[70] Kania A., Berent K., Mazur T., Sikora M. (2021). 3D printed composites with uniform distribution of Fe_3_O_4_ nanoparticles and magnetic shape anisotropy. Addit. Manuf..

[71] Aumnate C., Pongwisuthiruchte A., Pattananuwat P., Potiyaraj P. (2018). Fabrication of ABS/Graphene oxide composite filament for fused filament fabrication (FFF) 3D Printing. Adv. Mater. Sci. Eng..

[72] Hwang S., Reyes E.I., Moon K., Rumpf R.C., Kim N.S. (2015). Thermo-mechanical characterization of metal/polymer composite filaments and printing parameter study for fused deposition modeling in the 3D printing process. J. Electron. Mater..

[73] Kwok S.W., Goh K.H.H., Tan Z.D., Tan S.T.M., Tjiu W.W., Soh J.Y., Ng Z.J.G., Chan Y.Z., Hui H.K., Goh K.E.J. (2017). Electrically conductive filament for 3D-printed circuits and sensors. Appl. Mater. Today.

[74] Dickson A.N., Barry J.N., McDonnell K.A., Dowling D.P. (2017). Fabrication of continuous carbon, glass and Kevlar fibre reinforced polymer composites using additive manufacturing. Addit. Manuf..

[75] Carneiro O.S., Silva A.F., Gomes R. (2015). Fused deposition modeling with polypropylene. Mater. Des..

[76] Hamidi A., Tadesse Y. (2019). Single step 3D printing of bioinspired structures via metal reinforced thermoplastic and highly stretchable elastomer. Compos. Struct..

[77] Berretta S., Davies R., Shyng Y.T., Wang Y., Ghita O. (2017). Fused deposition modelling of high temperature polymers: Exploring CNT PEEK composites. Polym. Test..

[78] Bayraktar I., Doganay D., Coskun S., Kaynak C., Akca G., Unalan H.E. (2019). 3D printed antibacterial silver nanowire/polylactide nanocomposites. Compos. Part B Eng..

[79] Romani A., Mantelli A., Tralli P., Turri S., Levi M., Suriano R. (2021). Metallization of thermoplastic polymers and composites 3D printed by fused filament fabrication. Technologies.

[80] Romero P.E., Arribas-Barrios J., Rodriguez-Alabanda O., González-Merino R., Guerrero-Vaca G. (2021). Manufacture of polyurethane foam parts for automotive industry using FDM 3D printed molds. CIRP J. Manuf. Sci. Technol..

[81] Penumakala P.K., Santo J., Thomas A. (2020). A critical review on the fused deposition modeling of thermoplastic polymer composites. Compos. Part B Eng..

[82] Ahlhelm M., Günther P., Scheithauer U., Schwarzer E., Günther A., Slawik T., Moritz T., Michaelis A. (2016). Innovative and novel manufacturing methods of ceramics and metal-ceramic composites for biomedical applications. J. Eur. Ceram. Soc..

[83] Postiglione G., Natale G., Griffini G., Levi M., Turri S. (2015). Conductive 3D microstructures by direct 3D printing of polymer/carbon nanotube nanocomposites via liquid deposition modeling. Compos. Part A Appl. Sci. Manuf..

[84] Zhang P., Wang Z., Li J., Li X., Cheng L. (2020). From materials to devices using fused deposition modeling: A state-of-art review. Nanotechnol. Rev..

[85] Bardot M., Schulz M.D. (2020). Biodegradable poly(Lactic acid) nanocomposites for fused deposition modeling 3d printing. Nanomaterials.

[86] Silva M., Gomes C., Pinho I., Gonçalves H., Vale A.C., Covas J.A., Alves N.M., Paiva M.C. (2021). Poly(lactic acid)/graphite nanoplatelet nanocomposite filaments for ligament scaffolds. Nanomaterials.

[87] Cobos C.M., Fenollar O., López Martinez J., Ferrandiz S., Garzón L. (2020). Effect of maleinized linseed oil (MLO) on thermal and rheolological properties of PLA/MWCNT and PLA/HNT nanocomposites for additive manufacturing. Rapid Prototyp. J..

[88] Jing J., Chen Y., Shi S., Yang L., Lambin P. (2020). Facile and scalable fabrication of highly thermal conductive polyethylene/graphene nanocomposites by combining solid-state shear milling and FDM 3D-printing aligning methods. Chem. Eng. J..

[89] Dul S., Fambri L., Pegoretti A. (2020). Development of New Nanocomposites for 3D Printing Applications.

[90] Pezzana L., Riccucci G., Spriano S., Battegazzore D., Sangermano M., Chiappone A. (2021). 3d printing of pdms-like polymer nanocomposites with enhanced thermal conductivity: Boron nitride based photocuring system. Nanomaterials.

[91] Ferretti P., Santi G.M., Leon-cardenas C., Freddi M., Donnici G., Frizziero L., Liverani A. (2021). Molds with advanced materials for carbon fiber manufacturing with 3D printing technology. Polymers.

[92] Mantelli A., Romani A., Suriano R., Diani M., Colledani M., Sarlin E., Turri S., Levi M. (2021). Uv-assisted 3D printing of polymer composites from thermally and mechanically recycled carbon fibers. Polymers.

[93] Loh G.H., Sotayo A., Pei E. (2021). Development and testing of material extrusion additive manufactured polymer–textile composites. Fash. Text..

[94] Romani A., Rognoli V., Levi M. (2021). Design, materials, and extrusion-based additive manufacturing in circular economy contexts: From waste to new products. Sustainabiliy.

[95] Gkartzou E., Koumoulos E.P., Charitidis C.A. (2017). Production and 3D printing processing of bio-based thermoplastic filament. Manuf. Rev..

[96] Daver F., Lee K.P.M., Brandt M., Shanks R. (2018). Cork–PLA composite filaments for fused deposition modelling. Compos. Sci. Technol..

[97] Kariz M., Sernek M., Kuzman M.K. (2018). Effect of humidity on 3D-printed specimens from wood-pla filaments. Wood Res..

[98] Le Guen M.J., Hill S., Smith D., Theobald B., Gaugler E., Barakat A., Mayer-Laigle C. (2019). Influence of rice husk and wood biomass properties on the manufacture of filaments for fused deposition modeling. Front. Chem..

[99] Zhao D., Cai X., Shou G., Gu Y., Wang P. (2016). Study on the preparation of bamboo plastic composite intend for additive manufacturing. Key Eng. Mater..

[100] Stoof D., Pickering K., Zhang Y. (2017). Fused deposition modelling of natural fibre/polylactic acid composites. J. Compos. Sci..

[101] Osman M.A., Atia M.R.A. (2018). Investigation of ABS-rice straw composite feedstock filament for FDM. Rapid Prototyp. J..

[102] Tao Y., Wang H., Li Z., Li P., Shi S.Q. (2017). Development and application ofwood flour-filled polylactic acid composite filament for 3d printing. Materials.

[103] Depuydt D., Balthazar M., Hendrickx K., Six W., Ferraris E., Desplentere F., Ivens J., Van Vuure A.W. (2019). Production and characterization of bamboo and flax fiber reinforced polylactic acid filaments for fused deposition modeling (FDM). Polym. Compos..

[104] Bi H., Ren Z., Guo R., Xu M., Song Y. (2018). Fabrication of flexible wood flour/thermoplastic polyurethane elastomer composites using fused deposition molding. Ind. Crops Prod..

[105] Garcia Gonzalez M.N., Levi M., Turri S., Griffini G. (2017). Lignin nanoparticles by ultrasonication and their incorporation in waterborne polymer nanocomposites. J. Appl. Polym. Sci..

[106] Das A.K., Agar D.A., Rudolfsson M., Larsson S.H. (2021). A review on wood powders in 3D printing: Processes, properties and potential applications. J. Mater. Res. Technol..

[107] Henke K., Treml S. (2013). Wood based bulk material in 3D printing processes for applications in construction. Eur. J. Wood Wood Prod..

[108] Sia C.V., Nakai Y., Tanaka H., Shiozawa D. (2014). Interfacial fracture toughness evaluation of poly(L-lactide acid)/natural fiber composite by using double shear test method. Open J. Compos. Mater..

[109] Shinoj S., Visvanathan R., Panigrahi S., Kochubabu M. (2011). Oil palm fiber (OPF) and its composites: A review. Ind. Crops Prod..

[110] Tenhunen T.M., Moslemian O., Kammiovirta K., Harlin A., Kääriäinen P., Österberg M., Tammelin T., Orelma H. (2018). Surface tailoring and design-driven prototyping of fabrics with 3D-printing: An all-cellulose approach. Mater. Des..

[111] Yu S., Bale H., Park S., Hwang J.Y., Hong S.H. (2021). Anisotropic microstructure dependent mechanical behavior of 3D-printed basalt fiber-reinforced thermoplastic composites. Compos. Part B Eng..

[112] Ferrari F., Corcione C.E., Montagna F., Maffezzoli A. (2020). 3D printing of polymer waste for improving people’s awareness about marine litter. Polymers.

[113] Farina I., Singh N., Colangelo F., Luciano R., Bonazzi G., Fraternali F. (2019). High-performance Nylon-6 sustainable filaments for additive manufacturing. Materials.

[114] Wei B., Yang S., Wang Q. (2021). Green recycling of aluminum plastic packaging waste by solid-state shear milling and 3D printing for thermal conductive composites. Polym. Adv. Technol..

[115] John M.J., Dyanti N., Mokhena T., Agbakoba V., Sithole B. (2021). Design and development of cellulosic bionanocomposites from forestry waste residues for 3d printing applications. Materials.

[116] Figueroa-Velarde V., Diaz-Vidal T., Cisneros-López E.O., Robledo-Ortiz J.R., López-Naranjo E.J., Ortega-Gudiño P., Rosales-Rivera L.C. (2021). Mechanical and physicochemical properties of 3d-printed agave fibers/poly(Lactic) acid biocomposites. Materials.

[117] Rahimizadeh A., Kalman J., Fayazbakhsh K., Lessard L. (2021). Mechanical and thermal study of 3D printing composite filaments from wind turbine waste. Polym. Compos..

[118] Lendvai L., Singh T., Fekete G., Patnaik A., Dogossy G. (2021). Utilization of waste marble dust in poly(lactic acid)-based biocomposites: Mechanical, thermal and wear properties. J. Polym. Environ..

[119] Esposito Corcione C., Palumbo E., Masciullo A., Montagna F., Torricelli M.C. (2018). Fused deposition modeling (FDM): An innovative technique aimed at reusing Lecce stone waste for industrial design and building applications. Constr. Build. Mater..

[120] Tao Y., Liu M., Han W., Li P. (2021). Waste office paper filled polylactic acid composite filaments for 3D printing. Compos. Part B Eng..

[121] Tran T.N., Bayer I.S., Heredia-Guerrero J.A., Frugone M., Lagomarsino M., Maggio F., Athanassiou A. (2017). Cocoa shell waste biofilaments for 3D printing applications. Macromol. Mater. Eng..

[122] Kuo C.C., Wang C.W., Lee Y.F., Liu Y.L., Qiu Q.Y. (2017). A surface quality improvement apparatus for ABS parts fabricated by additive manufacturing. Int. J. Adv. Manuf. Technol..

[123] Klippstein H., Diaz De Cerio Sanchez A., Hassanin H., Zweiri Y., Seneviratne L. (2018). Fused deposition modeling for unmanned aerial vehicles (UAVs): A review. Adv. Eng. Mater..

[124] Uriondo A., Esperon-Miguez M., Perinpanayagam S. (2015). The present and future of additive manufacturing in the aerospace sector: A review of important aspects. Proc. Inst. Mech. Eng. Part G J. Aerosp. Eng..

[125] Kumar P., Rajak D.K., Abubakar M., Ali S.G.M., Hussain M. (2021). 3D Printing technology for biomedical practice: A review. J. Mater. Eng. Perform..

[126] Calcagnile P., Cacciatore G., Demitri C., Montagna F., Corcione C.E. (2018). A feasibility study of processing polydimethylsiloxane-sodium carboxymethylcellulose composites by a low-cost fused deposition modeling 3D printer. Materials.

[127] Aydin A., Demirtas Z., Ok M., Erkus H., Cebi G., Uysal E., Gunduz O., Ustundag C.B. (2021). 3D printing in the battle against COVID-19. Emergent Mater..

[128] Biswas M.C. (2019). Fused deposition modeling 3D printing technology in textile and fashion industry: Materials and innovation. Mod. Concepts Mater. Sci..

[129] Bhasin S., Singari R.M., Kumar H. (2021). Effect of 3D Printing on textile fabric. Advances in Manufacturing and Industrial Engineering.

[130] Cangelli E., Conteduca M. (2018). Architecture on demand. New scenarios for the design project and the construction industry. Techne.

[131] Singh S., Singh G., Prakash C., Ramakrishna S. (2020). Current status and future directions of fused filament fabrication. J. Manuf. Process..

[132] Greggio I. (2018). La stampa 3D nei Beni Culturali Analisi e Caratterizzazione di Materiali per la Fabbricazione Digitale di Beni Culturali. http://dspace.unive.it/bitstream/handle/10579/12795/843468-1216022.pdf?sequence=2.

[133] Chatterjee A., Dhande S.G. (2011). Heritage preservation in digital way—A contemporary research issue. Multimedia Information Extraction and Digital Heritage Preservation.

[134] Bonora V., Tucci G., Meucci A., Pagnini B. (2021). Photogrammetry and 3D printing for marble statues replicas: Critical issues and assessment. Sustainability.

[135] Scopigno R., Cignoni P., Pietroni N., Callieri M., Dellepiane M. (2017). Digital fabrication techniques for cultural heritage: A Survey. Comput. Graph. Forum.

[136] Nagy E.E., Nagy E.E. (2021). Studies in conservation the making of mike kelley’ s the wages of sin’ s exhibition copy: Replication as a means of preservation the making of Mike Kelley’s The Wages of Sin’ s exhibition copy: Replication as a means of preservation. Stud. Conserv..

[137] Ballarin M., Balletti C., Vernier P., Heritage C. (2020). Replicas in cultural heritage: 3D printing and the museum experience. Int. Arch. Photogramm. Remote Sens. Spat. Inf. Sci..

[138] Deng L., He K., Zhou T. (2020). Design of robotic and additive manufacturing for cultural heritage. IOP Conference Series: Materials Science and Engineering.

[139] Betocchi U., Madeddu N. (2016). Stampa 3D: Una nuova risorsa per gli allestimenti museali. Museol. Sci. Mem..

[140] Montusiewicz J., Barszcz M., Dziedzic K., Nowicki T. (2021). The method of decomposition of architectural objects for the preparation of 3D virtual models and replication. Adv. Sci. Technol. Res. J..

[141] Fotia A., Modafferi A., Nunnari A., Amico D., Feo L., Calabria R. (2021). From UAV survey to 3D printing, geomatics techniques for the enhancement of small village Cultural Heritage. J. WSEAS Trans. Environ. Dev..

[142] Monno A. (2010). Tecnologie 3D per i musei. Museol. Sci. Nuova Ser..

[143] Fiorenza L., Adams J.W., Yong R., Ranjitkar S., Hughes T., Quayle M., Mcmenamin P.G., Kaidonis J., Townsend G.C. (2018). Technical note: The use of 3D printing in dental anthropology collections. Am. J. Phys. Anthro..

[144] Higueras M., Calero A.I., Jos F. (2021). Digital 3D modeling using photogrammetry and 3D printing applied to the restoration of a Hispano-Roman architectural ornament. Digit. Appl. Archaeol. Cult. Herit..

[145] Fatuzzo G., Sequenzia G., Oliveri S.M., Barbagallo R., Calì M. (2017). An integrated approach to customize the packaging of heritage Artefacts. Lect. Notes Mech. Eng..

[146] Sánchez-Belenguer C., Vendrell-Vidal E., Sánchez-López M., Díaz-Marín C., Aura-Castro E. (2015). Automatic Production of Tailored Packaging for Fragile Archaeological Artifacts. J. Comput. Cult. Herit..

[147] Cronin C. (2021). Intellectual Property Implications of 3D Printing of Cultural Heritage 2010. https://papers.ssrn.com/sol3/papers.cfm?abstract_id=3849013.

[148] Scianna A., Filippo G. (2019). Di Rapid prototyping for the extension of the accessibility to cultural heritage for blind people. Int. Arch. Photogramm. Remote Sens. Spat. Inf. Sci..

[149] Auricchio F., Greco A., Alaimo G., Giacometti V., Marconi S. (2017). 3D printing technology for buildings’ accessibility: The tactile map for MTE Museum in Pavia. J. Civ. Eng. Archit.

[150] Anastasiadou C., Vettese S. (2019). “From souvenirs to 3D printed souvenirs”. Exploring the capabilities of additive manufacturing technologies in (re)-framing tourist souvenirs. Tour. Manag..

[151] Neumüller M., Reichinger A., Rist F., Kern C. (2014). 3D Printing for Cultural Heritage: Preservation, Accessibility, Research and Education Material Turn and Multi-Sensory Experiences in the Art.

[152] Balletti C., Ballarin M., Guerra F. (2017). 3D printing: State of the art and future perspectives. J. Cult. Herit..

[153] Scopigno R., Cignoni P., Pietroni N., Callieri M., Dellepiane M. Digital fabrication technologies for cultural heritage (STAR). Proceedings of the Eurographics Workshops on Graphics and Cultural Heritage.

[154] Heritage C., Martino S., Toolbox P.P. (2021). 3D Printing Applied to Cultural Heritage. https://www.digitalmeetsculture.net/article/3d-printing-applied-to-cultural-heritage/%3Fupm_export%3Dpdf+&cd=1&hl=it&ct=clnk&gl=it.

[155] Clini P., Mehtedi M.E., Nespeca R., Ruggeri L., Raffaelli E. (2017). A Digital reconstruction procedure from laser scanner survey to 3D printing: The theoretical model of The Arch of Trajan (Ancona). ScirestIT.

